# Class prediction for high-dimensional class-imbalanced data

**DOI:** 10.1186/1471-2105-11-523

**Published:** 2010-10-20

**Authors:** Rok Blagus, Lara Lusa

**Affiliations:** 1 Institute for Biostatistics and Medical Informatics, University of Ljubljana, Vrazov trg 2, Ljubljana, Slovenia

## Abstract

**Background:**

The goal of class prediction studies is to develop rules to accurately predict the class membership of new samples. The rules are derived using the values of the variables available for each subject: the main characteristic of high-dimensional data is that the number of variables greatly exceeds the number of samples. Frequently the classifiers are developed using class-imbalanced data, i.e., data sets where the number of samples in each class is not equal. Standard classification methods used on class-imbalanced data often produce classifiers that do not accurately predict the minority class; the prediction is biased towards the majority class. In this paper we investigate if the high-dimensionality poses additional challenges when dealing with class-imbalanced prediction. We evaluate the performance of six types of classifiers on class-imbalanced data, using simulated data and a publicly available data set from a breast cancer gene-expression microarray study. We also investigate the effectiveness of some strategies that are available to overcome the effect of class imbalance.

**Results:**

Our results show that the evaluated classifiers are highly sensitive to class imbalance and that variable selection introduces an additional bias towards classification into the majority class. Most new samples are assigned to the majority class from the training set, unless the difference between the classes is very large. As a consequence, the class-specific predictive accuracies differ considerably. When the class imbalance is not too severe, down-sizing and asymmetric bagging embedding variable selection work well, while over-sampling does not. Variable normalization can further worsen the performance of the classifiers.

**Conclusions:**

Our results show that matching the prevalence of the classes in training and test set does not guarantee good performance of classifiers and that the problems related to classification with class-imbalanced data are exacerbated when dealing with high-dimensional data. Researchers using class-imbalanced data should be careful in assessing the predictive accuracy of the classifiers and, unless the class imbalance is mild, they should always use an appropriate method for dealing with the class imbalance problem.

## Background

High-throughput technologies measure simultaneously tens of thousands of variables for each of the observations included in the study; data produced by these technologies are often called high-dimensional, because the number of variables greatly exceeds the number of observations. Microarrays are high-dimensional tools commonly used in the biomedical field; they measure the expression of genes [1] or miRNAs [2], the presence of DNA copy number alterations [3] or of variation at a single site in DNA [4], across the entire genome of a subject.

Microarrays are frequently used for class prediction (classification). In these studies the goal is to develop a rule based on the measurements (variables) obtained from the microarrays from samples (observations) that belong to distinct and well-defined groups (classes); these rules can be used to predict the class membership of new samples for which the values of the variables are known but the class-membership is unknown. For example, many studies tried to predict the clinical outcome of breast cancer using gene-expression [5]; in this case the classes are the clinical outcome of breast cancer while the variables are the expression of the genes. Some of the classification methods most frequently used for microarray data are discriminant analysis methods, nearest neighbor (k-NN, [6]) and nearest centroid classifiers [7], classification trees [8], random forests (RF, [9]) and support vector machines (SVM, [10]) (see [11] or [12] for an introduction to these methods).

An important aspect that specifically characterizes classification for high-dimensional data is the need to perform some type of variable selection. Variable selection consists in the identification of a subset of variables that are used to define the classification rule, and it can be performed either before developing the classifier or it can be embedded in the classification method [13]. The importance of variable selection for high-dimensional data rests on two facts: some classification rules cannot be derived if the number of variables is larger than the number of observations, and removing the variables that have little variability across observations improves the predictive accuracy [14].

In this paper we focus on classification problems for class-imbalanced data, i.e., on data sets where the number of observations belonging to each class is not the same. Class-imbalanced data are common in the biomedical field and they also arise when data are high-dimensional. For example, using gene-expression microarray data, Ramaswamy et al. [15] classified primary versus metastatic adenocarcinomas: metastatic specimen comprised about 16% of the training set (64 versus 12 samples); Shipp et al. [16] developed a classifier to distinguish diffuse large B-cell lymphoma from follicular lymphoma using a data set with a 25% class imbalance (58 versus 19 samples); IIzuka et al. [17] predicted early intrahepatic recurrence or non-recurrence for patients with hepatocellular carcinoma, with a training set with a 36% class imbalance (12 versus 21 samples). The classification methods used by these studies were some variants of the diagonal linear discriminant analysis (DLDA); the third study used also support vector machines.

Standard classification methods applied to class-imbalanced data often produce classification rules that do not accurately predict the minority class [18]; for this reason the between-class imbalance problem has been receiving increasing attention in recent years and many different strategies were proposed for deriving classification rules for imbalanced data (see [19] for a review). However, their use is not widespread in practice and very often standard classification methods are used when the classes are imbalanced [20]. For example, Ramaswamy et al. [15] and Shipp et al. [16] did not modify the classification rules to take class imbalance into account, while IIzuka et al. [17] tried to adjust for by making training and test set equally imbalanced.

The aim of our study was to investigate how class imbalance affects classification for high-dimensional data, and to evaluate if the high-dimensionality poses additional challenges when dealing with class-imbalanced data. We devoted special attention to the isolation of the possible effect of variable selection and to the investigation of the effectiveness of some strategies that were proposed to deal with class imbalance. To our knowledge the joint effect of high-dimensionality and class imbalance on classification has not been thoroughly investigated.

The few works that dealt with the class imbalance problem for high-dimensional data mostly focused on developing methods for variable selection [21], on the comparison of the performance of classifiers using different variable selection methods and/or classifiers [21-23], or on proposing and evaluating different strategies for adjusting classifiers trained on class-imbalanced data [24-27].

To investigate the effect of class imbalance on high-dimensional data, we evaluated the performance of six types of classifiers on imbalanced data. The classification methods were chosen among those most commonly used for high-dimensional data and for the sake of simplicity we considered only classification problems where the number of classes was two (two-class classification problems). The classifiers were evaluated both on simulated data and on a publicly available data set from a breast cancer gene expression microarray study [28]; we assessed both the overall and the class specific predictive accuracy of the classifiers. We simulated situations where there was no difference between classes (null case) and where the two classes were different (alternative case), varying the number of different variables and the magnitude of their difference. We used over-sampling, down-sizing and a variant of asymmetric bagging to correct the class imbalance problem.

In Results we present a series of selected simulation studies showing the consequences of using class-imbalanced high-dimensional data sets for classification, we show the performance of the corrections for class imbalance, and the results obtained on the breast cancer data. In Discussion we outline the problems related to classification for high-dimensional data. In the Methods section we briefly describe the classification methods that we used and the strategies to deal with the class imbalance problem; we also describe the simulations that were performed and the breast cancer gene expression microarray data.

## Results

The classifiers were developed on the training sets, while the predictive accuracy (PA, overall and class specific: PA_1 _for Class 1 and PA_2 _for Class 2), predictive values (PV_1 _and PV_2_) and area under the ROC curve (AUC) were evaluated on the test sets. If not otherwise stated, the samples were normalized (mean-centered), while the variables were not (see Methods), and the test sets were balanced ($k_{1}^{test}$ = 0.5). The classification with RF and penalized logistic regression (PLR) were based on the 0.5 threshold, if not differently specified (see Methods). Each simulation was repeated 1000 times. Most of the figures show the results only for DLDA, PLR and one of the nearest neighbor classifier; the results for the other classifiers are shown in the Additional Files.

### Simulations results: Null case

Under the null case there was no difference between the two classes, as all the variables were simulated from the same distribution (see Methods for details on data simulation). In the first set of simulations only *p *= 40 variables were generated, and all were used to derive the classification rule (*G *= *p*). The imbalance was the same in the training and in the test set ($k_{1}^{train}=k_{1}^{test}$).

**Figure 1:**
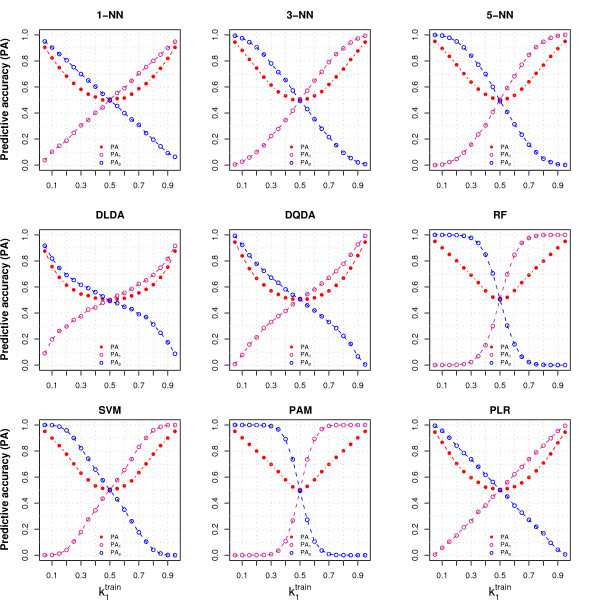
**Behavior of the classifiers under the null hypothesis with no variable selection**. The figure shows the overall (PA) and class specific predictive accuracies (PA_1 _and PA_2_), varying the proportion of samples from Class 1 in the training set ($k_{1}^{train}$), for nine classifiers. The training set contained 80 samples, and *p *= 40 variables were generated from the same distribution for both classes (*N*(0, 1)); no variable selection was performed (*G *= *p*). The proportion of Class 1 samples in the test set, containing 20 samples, was the same as in the training set. The PA were evaluated on the test set. Samples were mean centered. Details on data generation and on classifiers are reported in the Methods section.

The class specific PA were not equal when the classes were imbalanced: most of the samples from the test set were classified in the majority class, which had a larger PA compared to the minority class (Figure 1 and Additional File 1). However, in all the situations the classifier showed no relation with the outcome (PA_1 _= 1 - PA_2_) and had no information about the true class status (PV_1 _= 1 - PV_2 _= *κ*_1_, the assumed proportion of samples from Class 1 in the population, and AUC = 0.5, Additional File 1).

The overall PA reached its minimum value when the data were balanced (PA = 0.5), and increased when the class imbalance of the test set became larger (Figure 1 and Eq. 5). The average class specific PA depended on the class imbalance of the training set but not on the class imbalance of the test set (Additional File 1); moreover, the overall PA was equal to 0.5 for all the classifiers when the test set was balanced, regardless of the imbalance of the training set (Additional File 1 and Eq. 5).

**Figure 2:**
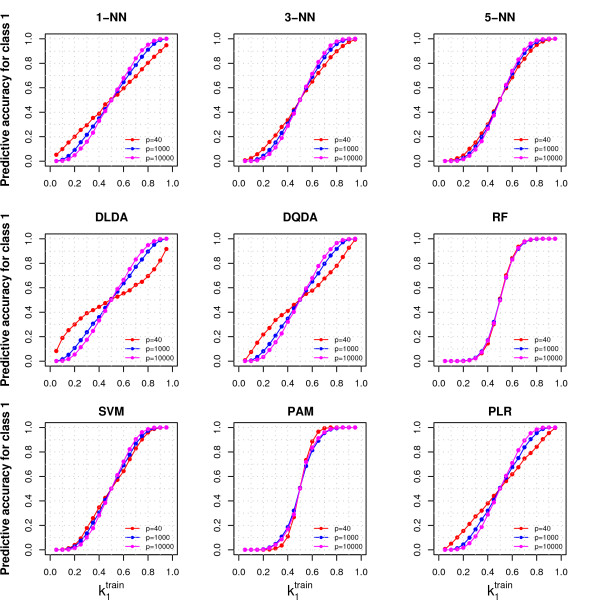
**Effect of variable selection under the null hypothesis**. The figure shows the Class 1 predictive accuracy (PA_1_) obtained varying the proportion of samples from Class 1 in the training set ($k_{1}^{train}$), using nine classifiers. The training set contained 80 samples, and *p *= 40, 1000 or 10000 variables were generated from the same distribution for both classes (*N*(0, 1)); 40 variables were selected (*G *= 40). The test set was balanced ($k_{1}^{test}$ = 0.5) and contained 20 samples. The PA were evaluated on the test set. Samples were mean centered. Details on data generation are reported in the Methods section.

For most classifiers, performing variable selection further increased the probability of classifying a new sample in the training set majority class (Figure 2). For example, for 1-NN with $k_{1}^{train}$ = 0.1, when we increased the number of variables (*p *= 1000 and 10000) and performed variable selection (*G *= 40) we obtained PA_1 _= 0.02 and 0.00, and PA_2 _= 0.98 and 1.00, respectively, instead of the values expected under the null case (0.1 and 0.9, the proportion of samples from each class in the training set); also in this case the classifiers were not informative (PA_1 _= 1 - PA_2_). The departures of the PA from the expected values depended on the procedure of variable selection, as we did not observe a similar effect when we increased the number of variables (*p *= 1000 and 10000) but did not perform variable selection (*G *= *p*).

The effect of variable selection can be explained recognizing that the sampling variability is larger in the minority class. Sample mean values far from the true population values arise more frequently in the minority class, and the variables that show large differences between the classes are more likely to be selected. The new samples from the test set are therefore more similar to the samples of the majority class, and as a consequence they have a larger probability of being classified in that class. We observed this behavior not only for t-test with equal variances but also for other commonly used parametric and non-parametric variable selection methods (Additional File 2).

Among the classifiers that we considered, RF, SVM and PAM (Prediction Analysis of Microarrays) were the most sensitive to class imbalance when we did not perform variable selection (showing the largest difference between class-specific PA, Figure 1), while apparently variable selection had little or no effect on their class-specific PA (Figure 2). The reason is that these classifiers perform some type of variable selection automatically, therefore for these classifiers the results of Figure 1 embed variable selection. When the classification rules of RF and PLR were adjusted to take the class imbalance into account (RF-THR and PLR-THR, see Methods), the dependency of the class specific PA on class-imblance diminished but it did not disappear (data not shown). Variable normalization (see Methods) did not change the null case results: regardless of the class imbalance, its impact on data was very limited since the true means of all variables were all equal (data not shown).

### Simulation results: Alternative case

For the alternative case we considered situations in which some of the variables had different means in the two classes, varying the number of different variables (*p_DE_*) and the mean difference (*μ*^(2)^) (see Methods for details).

**Figure 3:**
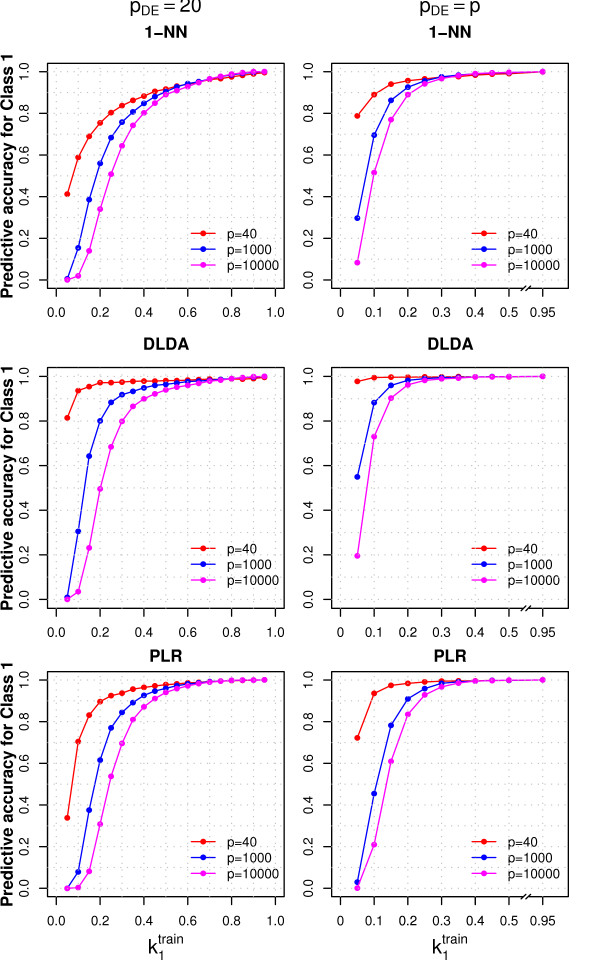
**Effect of performing variable selection and increasing the number of variables**. The figure shows the predictive accuracy for Class 1 (PA_1_), varying the proportion of samples from Class 1 in the training set ($k_{1}^{train}$), for three classifiers. Forty, 1000 or 10000 variables (*p*) were generated and 40 variables were selected and used to develop the classifiers. In the left panels the mean of *p_DE _*= 20 variables was different in the two classes, while in the right panels all the variables had different means (see Methods for details on data generation). The training set contained 80 samples, while the test set contained 20 samples and was balanced. Additional file 4 shows the results for all the classifiers.

Similarly to the null case, most samples were classified in the majority class: the class specific PA of the minority class rapidly decreased as the class imbalance increased; this effect was more marked when the differences between classes were smaller (data not shown) or when we increased the number of variables (from *p *= 40 to *p *= 1000 and 10000) and performed variable selection (Figure 3, left panels, where *p_DE _*= 20, *μ*^(2) ^= 1 and *G *= 40 variables were selected). The AUC and PV of the majority class decreased as the class imbalance increased, even though not substantially (Additional File 3): the limited decrease of the PV of the majority class can be explained recalling that under the null hypothesis its value is large, being equal to the proportion of samples from that class in the population (PV_*i *_= *κ_i_*). The PV of the minority class increased (Additional File 3): when the PA of one class approaches the value of 1, so does the PV of the other class (Eq. 6). The dependency of PV and AUC on class imbalance was more marked if smaller differences between classes were considered (data not shown).

The noise introduced in the classifier by selecting null-variables was only partially responsible for the decrease in the PA of the minority class (observed when the number of variables was increased). In an attenuated form this effect was still present even when all the variables were different between the two classes (*p_DE _*= *p*, Figure 3, right panels). Similarly to the null case, we were more likely to select variables for which the discrepancy between the true and the sample values was larger in the minority class; as a consequence we were less likely to classify new samples in this class. This behavior was not a peculiarity of the t-test with equal variances, but was observed also for the other variable selection methods that we considered (Additional File 2).

The classifier that showed the smallest decrease in the PA for the minority class was DLDA, which was practically insensitive to class imbalance when the number of variables was small (*p *= 40 and *p_DE _*= 20, 40, Figure 3); PAM, SVM and RF were the most sensitive to class imbalance also under the alternative case (Additional File 4 and 5).

Similarly to previous findings [14] we also observed that variable selection improved the performance of the classifiers under the alternative case: the class specific PA were consistently better when variable selection was performed, also for situations where there was a large class imbalance (see Additional File 6 for results where all the variables were included in the classifiers).

**Figure 4:**
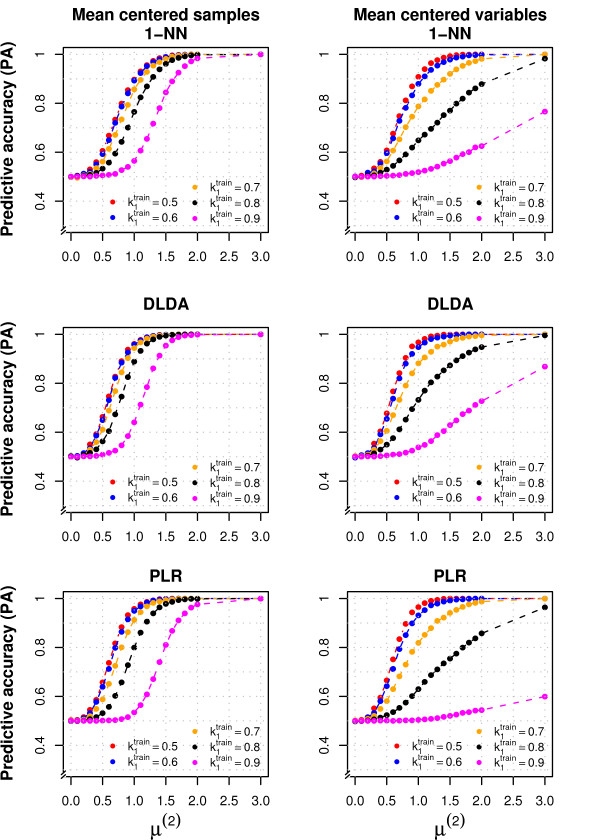
**Performance of three classifiers varying the between class differences and normalization methods**. The figure shows the overall predictive accuracy (PA) for 1-NN, DLDA and PLR, obtained varying the difference between classes. We considered the same simulation settings of the left panels of Fig. 3 (p = 1000) but varied the mean of the *p_DE _*= 20 non-null variables (*μ*^(2)^). The results obtained by normalizing samples (left panels) or variables (right panels) are reported. Details on data generation are reported in the Methods section.

We evaluated how the performance of the classifiers was affected by the magnitude of the difference between classes (Figure 4): we considered the same simulation settings of Figure 3 but varied the mean of the *p_DE _*= 20 non-null variables (*μ*^(2) ^= 0, 0.25, 0.5, ..., 2, 2.5, 3). For the balanced training set ($k_{1}^{train}$ = 0.5) the overall PA (Figure 4, left panels) reached the value of 1 when *μ*^(2) ^was between 1 and 1.5 for all the classifiers; much larger differences between the classes were needed (*μ*^(2) ^≥ 2) to obtain the same PA for the highly imbalanced data ($k_{1}^{train}$ = 0.1). The differences between classifiers trained with different class imbalance were not entirely due to their ability of selecting the non-null variables (all the non-null variables were selected in almost all simulations when *μ*^(2) ^≥1.5). RF, SVM and PAM required the greatest difference between classes in order to predict correctly all the samples in the imbalanced cases (Additional File 7). Normalizing the variables worsened the PA on class-imbalanced data (Figure 4, right panels); this can be attributed to the different class imbalance in training and test set ($k_{1}^{test}$ = 0.50), as variable normalization did not have this negative effect when the imbalance was the same (data not shown).

### Solutions

All the solutions were evaluated using the same simulation settings described for Figure 3, left panels, with *p *= 1000.

#### Over-sampling

**Figure 5:**
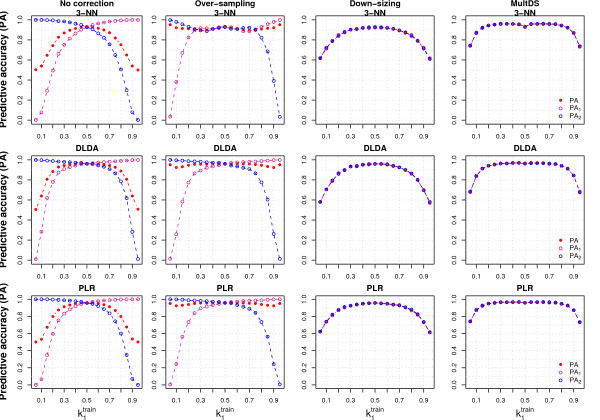
**Solutions to the class imbalance problem**. The figure shows, for three classifiers, the overall (PA) and class specific predictive accuracies (PA_1 _and PA_2_), varying the proportion of Class 1 samples in the training set ($k_{1}^{train}$). The simulation settings are the same as those from the left panels of Fig. 3 (*p *= 1000). The results are given for the situations when no class imbalance correction is applied (No correction, first column), or when over-sampling (second column), down-sizing (third column) or multiple down-sizing (MultDS, forth column) are used.

We ran a set of simulations in which we obtained a balanced training set by increasing the sample size, replicating the samples from the minority class (over-sampling, see Methods for details). Over-sampling (Figure 5 and Additional File 8, second column) produced almost no change in PA compared to full-data analysis (Figure 5 and Additional File 8, first column) for most classifiers. Most of the classification rules were just slightly modified by the presence of replicated samples, as they depended on the within-class mean and variability of the variables, which are hardly modified by over-sampling. For the same reasons the variable selection process was also not substantially affected by over-sampling. Only 3-NN and 5-NN benefitted from over-sampling, while 1-NN was not modified at all (Additional File 8).

The performance of 1-NN, DLDA and PLR improved when over-sampling was used together with variable normalization, but only when the test set was balanced ($k_{1}^{test}$ = 0.5), therefore this result seems of limited practical utility (in Additional File 9 we give a possible explanation of this phenomenon for DLDA). Over-sampling with variable normalization partly removed the dependence of class specific PA on class imbalance for RF and PAM when there was the same imbalance in training and test set ($k_{1}^{test}=k_{1}^{train}$), however only if the class imbalance was moderate (0.30 ≤ $k_{1}^{train}$ ≤ 0.70, Additional File 9).

#### Down-sizing

In a second set of simulations we obtained a balanced training set by removing a subset of samples from the majority class (down-sizing, see Methods). The PA of the minority class was greatly improved by down-sizing and the class-specific PA became the same for both classes, regardless of the class imbalance in the original training set (Figure 5 and Additional File 8, third column). For example, using only 4 samples per class ($k_{1}^{train}$ = 0.1), all the classifiers achieved a PA of about 0.70 for both classes, while the full-data analysis assigned all the samples from the test set to the majority class (PA_1 _= 0 and PA_2 _= 1 using *n*_1 _= 4 and *n*_2 _= 76). The PV of the majority class further increased, while the PV of the minority class decreased substantially as the PA of the majority class moved away from 1 (see Eq. 6). The classifiers that were the most sensitive to class imbalance were those that benefitted the most from down-sizing. For example, PA_1 _increased from 0.5 (full-data) to 0.8 when we down-sized the training set using PAM with $k_{1}^{train}$ = 0.2 (Additional File 8).

Importantly, the variability of the estimated PA obtained by down-sizing increased with class imbalance; the 95% prediction intervals obtained for $k_{1}^{train}$ = 0.5 were between 0.8 and 1.0 while they were between 0.50 and 0.90 when $k_{1}^{train}$ = 0.10 (Additional File 10).

The PA (overall and class-specific) obtained by down-sizing decreased as the class imbalance increased (as $k_{1}^{train}$ moved away from 0.50); this effect was not due to class imbalance but to the decrease in sample size of the training set.

#### Multiple down-sizing (Asymmetric bagging with variable selection)

Neglecting information from the majority class as in simple down-sizing is intuitively unappealing, therefore we considered multiple down-sizing (MultDS), i.e., for each training set we repeatedly down-sized the training set, randomly selecting the samples from the majority class and including all the samples from the minority class, developed a classifiers on each training set, and assigned new samples to the class to which they were classified more frequently (see Methods for details). The performance of MultDS (Figure 5, forth column) was similar but consistently better than down-sizing in terms of average PA. The decrease of PA for imbalanced data due to smaller sample size was still present but less pronounced. In our simulation settings the PA of all classifiers did not vary for a wide range of imbalance levels ($k_{1}^{train}$ = 0.20 to 0.80). MultDS had a smaller variability of PA compared to simple down-sizing (Additional File 10); for example, when $k_{1}^{train}$ = 0.1 the average PA was around 0.85 for most classifiers and 95% prediction intervals were between 0.7 and 1.0.

To evaluate if the results obtained by MultDS were influenced by the number of samples left out to obtain a balanced training set we run a set of additional simulations where the sample size was smaller (*n_train _*= 50, *n_test _*= 20): at the same level of class imbalance MultDS worked better when the number of samples was larger; the observed differences increased with class imbalance and the performance of MultDS became similar to simple down-sizing when the sample size was small (data not shown).

#### Use of different threshold for RF and PLR

We considered two variants of RF and PLR, where the threshold value used for classification was based on the class imbalance of the training set rather than on the fixed value of 0.5 (RF-THR and PLR-THR, see Methods). Using these variants the dependency of the class specific PA on the class imbalance was less pronouced but still present (Additional File 11).

### An application to breast cancer microarray data

We used the published gene-expression microarray data set of Sotiriou et al. [28] to evaluate the effect of class imbalance on classification for real high-dimensional data (see Methods for details on data). We considered two classification problems: the prediction of estrogen receptor (ER) status (ER+ vs ER-) and of grade of breast cancer (1 or 2 vs 3).

We obtained different levels of class imbalance by repeatedly randomly selecting subsets of the samples from the complete data set: 500 different training and test sets were obtained for each situation.

**Figure 6:**
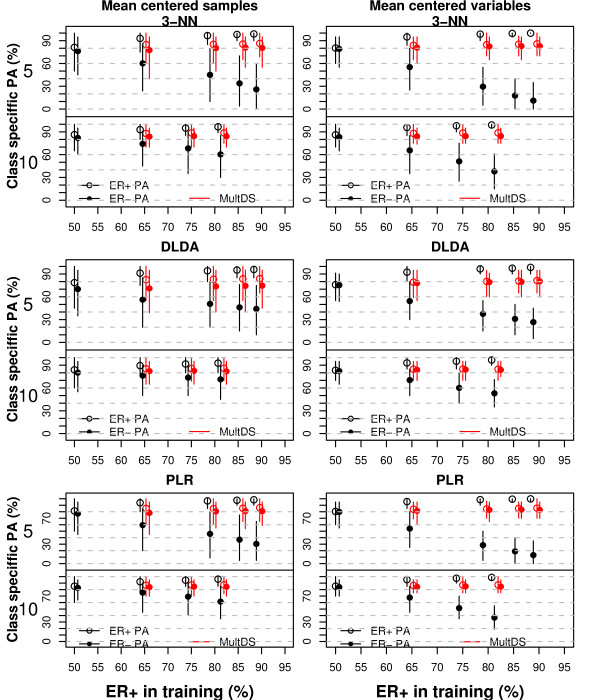
**Prediction of ER status**. The figure shows the class specific predictive accuracies (dots, white for the ER+ and colored for the ER-class; expressed as %), and their 95% prediction intervals. The results reported in black and white refer to simple down-sizing, while those in red to multiple down-sizing. Five ER- samples (upper panels) or 10 ER-samples were included in the training set, while the number of ER+ samples was 5, 10, 20, 30 or 45. The test sets were balanced (20 ER- and 20 ER+ samples). The samples (left panels) or the variables (right panels) were mean-centered.

In a first set of analyses we trained the classifiers using class balanced and class-imbalanced training sets (*n_ER_*_- _= 5 vs *n_ER_*_+ _= 5, 10, 20, 30, 45, and *n_ER- _*= 10 vs *n_ER_*_+ _= 10, 20, 30, 45); the test sets were balanced (20 ER+ vs 20 ER-). The class specific PA (PA_*ER*+ _and PA_*ER*-_) were strongly dependent on class imbalance of the training sets for all the classifiers (Figure 6 and Additional Files 12 and 13); most test set samples were classified in the ER+ class (majority class in the training set) and PA_*ER*+ _was larger than PA_*ER*-_. For example, when the training sets included 20 ER+ and 5 ER- samples, the PA_*ER*+ _was above 0.90 for all the classifiers, while PA_*ER- *_ranged from about 0.2 to 0.6; the PV for the ER+ class (PV_*ER*+_) was about 0.9, while it ranged between 0.7 and 0.8 for the ER- class (PV_*ER-*_), and the AUC was between 0.8 and 0.9.

Using the smaller but balanced training set (down-sizing) the class specific PA were approximately the same (about 0.75 and 0.85, using 5 or 10 samples per class, respectively). For most classifiers the AUC and PV_*ER*+ _were smaller than those obtained using the larger imbalaced data, while the PV_*ER- *_were larger. Similarly to the simulation studies, DLDA was the least sensitive to class imbalance. Over-sampling did not remove the dependency of class-specific PA on class imbalance for most of the classifiers, with differences between class-specific PA as large as 0.22 (20 ER+ vs 10 ER-, DQDA). Over-sampling worked reasonably well only for 3-NN and 5-NN when the imbalance was not too large (data not shown); these results were in line with the simulation studies results.

We used MultDS for several different levels of class imbalance in the training set (Figure 6 and Additional File 14). PAM seemed to benefit the most from MultDS; however, the gains in PA achieved by using MultDS rather than simple down-sizing were not as considerable as those observed in the simulation studies for the same level of class imbalance. This could be the consequence of using a smaller sample size in the breast cancer application.

The major advantage of MultDS over simple down-sizing was the reduction of variability of the estimated predictive accuracy; for example, when the minority class included only 5 samples (upper panels of Figure 6) the prediction intervals obtained by simple down-sizing included the value of 0.50 for all the classifiers, while the lower limit of the prediction intervals obtained by MultDS were above 0.60 for most classifiers even for the largest degree of class imbalance. Compared to simple down-sizing, the PV_*ER*+ _slightly increased, while the PV_*ER- *_decreased, ranging between 0.50 and 0.60; the AUC increased (Additional File 14).

Grade of breast cancer was more difficult to predict than ER status (overall PA about 0.60 using the small balanced training set and about 0.70 when using the larger training set, data not shown). The smaller differences in between-class gene-expression translated in larger sensitivity of the classifiers to class imbalance (PA for the minority class was between 0.10 and 0.20 when we used the most imbalanced data set), therefore the effect of class imbalance was stronger. Overall, the results obtained for grade prediction were in line with those obtained from the simulations.

Variable normalization further increased the effect of class imbalance, causing even more cases to be classified in the training set majority class (data not shown). Using normalized variables down-sizing worked well, and so did over-sampling for k-NN, DLDA and PLR, but only if the test set was balanced; therefore, the practical importance of this result seems very limited.

## Discussion

Our results showed that some of the classifiers that are more frequently used for class prediction with high-dimensional data are highly sensitive to class imbalance. All the classifiers that we considered assign most of the new samples to the majority class from the training set, unless the difference between classes is large. This problem arises for two reasons: the probability of assigning a new sample to a given class depends on the prevalence of that class in the training set, and with variable selection this probability is further biased towards the majority class. As a consequence, when classifiers are trained on class-imbalanced data there are usually large differences between class specific predictive accuracies; moreover, the overall predictive accuracy is not informative, especially when both the training and the test set are imbalanced ([29], chapter 2). In most circumstances, the unequal predictive accuracies produced by class-imbalanced classifiers have the effect of slightly decreasing the difference in the class-specific predictive values, which is present when the predictive accuracies are equal and the classes are imbalanced; generally, the predictive values of the minority class increase, while there is a slight reduction for those from the majority class, which are large even when the classifer is uninformative. Similarly to our previous findings for another classifier [30], we observed that also in this setting all these properties are mantained even if the prevalence in the training and test set is matched. Normalizing (centering) the variables generally additionally biases the classification results, and only if the training and test set have the same imbalance it does not produce an additional negative effect. Using the embedded class imbalance corrections available for RF, SVM or PAM does not remove the dependency of the classification probabilities on class imbalance. Our results indicate that variable selection further increases the probability of assigning a new sample to the majority class; the reason is that the sampling variability is larger in the minority class and therefore the biggest deviations between the true and the observed values arise in this class. As a consequence, the selected variables are those that have the biggest departures from the true values in the minority class, either indicating differences between classes that do not exist (null variables), or amplifying some differences that exist (non-null variables). However, at the same time variable selection plays also a positive role in high-dimensional classification; similarly to previous findings [14] our results also indicate that the predictive accuracy of all the classifiers is improved if the classification rule is derived using only a selected subset of the measured variables.

The next question is if there are satisfactory remedies for these problems. A first set of solutions consisted in creating a balanced training set, either by replicating (over-sampling) or by removing (down-sizing) some of the samples. Over-sampling does not remove or attenuate the class imbalance problem [31] also in our settings because we considered classification rules and a variable selection method that are hardly modified by the presence of replicated samples; the k-NN classifiers (*k *> 1) are the only exeption. On the other hand, simple down-sizing works well in removing the discrepancy between the class-specific predictive accuracies, but as expected it has a large variability and the predictive accuracy of the classifiers worsens when the effective sample size is reduced considerably because the class imbalance is large.

Our attempt in overcoming these problems was the combination of classifiers trained on balanced down-sized training set. Multiple down-sizing can be seen as a special case of asymmetric bagging [24], except that the variables are selected in each down-sized training set. We based the combination of the classifiers on majority voting even though more complex methods are available; for example, EasyEnsemble [32] combines the outputs from the classifiers using AdaBoost [33,34].

In practice multiple down-sizing improves on simple down-sizing: the major advantage is the reduction of variability of the estimated predictive accuracy [35]; in some situations we observed also a limited improvement of predictive accuracy. The relative benefit of multiple down-sizing over simple down-sizing depends on the amount of information discarded by simple down-sizing, i.e., on the level of class imbalance but also on the number of left-out samples. The real data had smaller sample size compared to the simulated data and for that reason multiple down-sizing was not as beneficial on real data as in the simulations.

We used penalized logistic regression (PLR) as a classification method and evaluated its predictive accuracy as the fraction of correctly classified samples (see the limitations of this approach in [36], page 247). The classification based on the 0.50 threshold on the predicted probabilities assigns most samples to the majority class from the training set, similarly to simple logistic regression. Using the threshold based on the imbalance from the training set, which works well for logistic regression, reduces but does not remove the classification bias towards the majority class, also when no variable selection is performed before fitting the PLR model; variable selection further increases this bias. Similar results hold for random forest.

We did not attempt to perform a comprehensive study on class imbalance for high-dimensional data, but we focused solely on some types of classifiers and of classification strategies. We selected which classifiers to evaluate among those that are most commonly used for high-dimensional data. It is possible that other methods that we did not consider might be less sensitive to class imbalance. Most of our results were based on one single method for variable selection, i.e., t-test with equal variances, which bases the selection of the genes on the difference between their means. Some of the effects of variable selection on class-imbalanced classification might depend on this choice. However, our results show that also other parametric and non-parametric variable selection methods have the same type of problem on imbalanced data. This is because they all attempt to select, among a very large number of candidate variables, those that differ the most between the classes, using different metrics to define the difference. We decided to focus on some of the solutions for the class imbalance problem: over-sampling, down-sizing and multiple down-sizing. We observed that these approaches performed well in removing the bias towards the classification into the majority class, with the exception of over-sampling. More complex methods might be more effective in reducing the variability of the predictive accuracy. The development of guidelines for the design of class prediction studies with class-imbalanced data is also a very important issue, which we considered only marginally in this paper.

## Conclusions

Our results show that the naive use of classifiers on class-imbalanced high-dimensional data can produce classification results that are highly biased towards the classification in the majority class. The extent of this bias depends on the classification method, on the magnitude of the difference between classes, and on the level of class imbalance, and it is further increased when variable selection methods are used; variable normalization generally increases the bias and it should be avoided, unless the class imbalance is equal in training and test set. When class imbalance is moderate, and no correction for class imbalance is applied, our results indicate that DLDA performs well. In addition to its relative robustness to class imbalance, another advantage of DLDA is its simplicity and interpretability.

Our results suggest that using a balanced training set is a good choice for the design of high-dimensional class prediction studies, also in situations where the proportion of samples from each class is not equal in the population. If class imbalance cannot be avoided, researchers should take the class imbalance problem into account, and appropriately adjust their classification rules. We showed that multiple down-sizing can be effectively used if class imbalance is not too severe. This method is useful in removing the bias towards the classification in the majority class, and in reducing the variability of the predictive accuracy compared to simple down-sizing. Further work is needed in order to asses if more complex approaches to the correction of class imbalance problem can further increase the class-specific predictive accuracies and predictive values, and reduce their variability.

## Methods

### Notation

Let *x_ij _*be the expression of *j *th variable (*j *= 1, ..., *p*) on *i*th individual (*i *= 1, ..., *n*). Some of the samples are known to belong to Class 1 (*n*_1 _samples) and others to Class 2 (*n*_2_). Let *κ_i_*, $k_{i}^{train}$ and $k_{i}^{test}$ denote the proportion of samples from Class *i *in the population, in the training set and in the test set, respectively. We limit our attention to the *G *variables (*G *≤ *p*) that are the most informative about the class distinction. We defined the most informative variables to be those with the largest absolute value of the univariate statistic derived from the two-sided t test with equal variances. For sample *i *we denote the set of selected variables by **x**_*i*_. Let ${\bar{x}}_{j}^{\left(1\right)}$ and ${\bar{x}}_{j}^{\left(2\right)}$ denote the mean expression of the *j*th selected variable in Classes 1 and 2, respectively. Let x* represent the set of selected variables for a new sample.

### Analysis

Statistical analysis and simulations were carried out using R language for statistical computing (R version 2.8.1) [37].

### Classification methods

#### *k*-nearest neighbors

Nearest neighbor rules (k-NN, [38]) are simple nonparametric methods that classify a new specimen based on the class labels of its nearest neighbors, i.e., of the specimens in the training set to which its variables are most similar. The class of the new specimen is predicted to be the majority class label of its *k *nearest neighbors [12]. In this paper we used the Euclidean distance to define the distance between samples and used 3 different k-NN classifiers, with *k *= 1, 3 and 5.

Analysis was performed with the knn function of the *class *package in R.

#### Discriminant analysis

Discriminant analysis methods are used to find linear combination of variables that maximize the between-class variance and at the same time minimize the within-class variance [11,12]. Special cases of discriminant analysis are diagonal linear discriminant analysis (DLDA) and diagonal quadratic discriminant analysis (DQDA). DLDA makes the assumption that the variables are independent and have the same variability in both classes.

DLDA classification rule assigns a new sample (with expression of the selected genes x*) to Class 1 if

(1) $$\sum_{g=1}^{G}\frac{{\left(x_{g}^{*}-{\bar{x}}_{g}^{\left(1\right)}\right)}^{2}}{s_{g}^{2}}\leq \sum_{g=1}^{G}\frac{{\left(x_{g}^{*}-{\bar{x}}_{g}^{\left(2\right)}\right)}^{2}}{s_{g}^{2}},$$

and to Class 2 otherwise. In Eq. 1 $s_{g}^{2}$ is the sample estimate of the pooled variance for variable *g*. DQDA assumes that the variables are independent but that their variances can be different in the two classes. DQDA classification rule assigns a new sample to Class 1 if

(2) $$\begin{array}{l} \sum_{g=1}^{G}\left(\frac{{\left(x_{g}^{*}-{\bar{x}}_{g}^{\left(1\right)}\right)}^{2}}{s_{1g}^{2}}+log(s_{1g}^{2})\right)\\ \leq \sum_{g=1}^{G}\left(\frac{{\left(x_{g}^{*}-{\bar{x}}_{g}^{\left(2\right)}\right)}^{2}}{s_{2g}^{2}}+log(s_{2g}^{2})\right), \end{array}$$

and to Class 2 otherwise. $s_{1g}^{2}$ and $s_{2g}^{2}$ are the estimated variances of variable *g *in Class 1 and Class 2, respectively.

The function stat.diag.da in the *sma *package was used to perform DLDA and DQDA.

#### Random forest

Random forest (RF, [39]) is a classifier consisting of an ensemble of classification trees; each of the *T *trees is built on a bootstrap sample drawn from the complete data, *m *among the *p *variables are randomly chosen and used to find the best split for each node, trees are not pruned, i.e., they are grown to the largest extent, and the new samples are assigned to a class for each of the trees. RF classifies the new samples in the class to which they were assigned most frequently by the *T *trees.

To take into account the class imbalance of the training set we considered also a different classifier, which assigned a new sample to Class 1 if the proportion of classification assignments to Class 1 was greater than the proportion of samples in Class 1 in the training set ($k_{1}^{train}$). This corrected classifier is referred in the text as RF-THR. We assessed the performance of both classification rules.

We used the function randomForest in the *randomForest *package, used default values for the parameters (*T *= 500, *m *= $\sqrt{p}$) and defined class priors equal to the proportion of samples in each class in the training set - option classwt. The RF-THR classification was obtained using the option cutoff in the randomForest function.

#### Support vector machines

Linear support vector machines (SVM; [40]) attempt to find a linear combination of the variables that best separates the samples into two groups based on their class labels. When perfect separation is not possible, the optimal linear combination is determined by a criterion to minimize the number of misclassifications and simultaneously maximize the distance between the classes.

In this paper we used the functions svm and predict from the *e1071 *package, with linear kernel basis, we specified the class weights as the proportion of samples from each class and did not use the default variable scaling of the svm function.

#### PAM

Nearest shrunken centroids classification (also known as "Prediction Analysis of Microarrays", PAM, [41]) is an enhancement of the simple nearest centroid classification, where a new sample is classified in the class whose centroid is closest to, in squared distance. The centroid of a class is the vector containing the mean expression profile of all samples from that class in the training set.

The shrunken centroids are obtained by standardizing the centroids (dividing the centroid value for each variable by the within-class standard deviation for that variable (*s_g_*)), and by shrinking each class centroid toward the overall centroid for all classes by an amount called threshold. A test sample x* is classified in Class 1 if

(3) $$\begin{array}{l} \left(\sum_{g=1}^{G}\frac{{\left(x_{g}^{*}-{\bar{x}}_{g}^{,\;(1)}\right)}^{2}}{{\left(s_{g}+s_{0}\right)}^{2}}-2log\pi_{1}\right)\\ \leq \left(\sum_{g=1}^{G}\frac{{\left(x_{g}^{*}-{\bar{x}}_{g}^{,\;(2)}\right)}^{2}}{{\left(s_{g}+s_{0}\right)}^{2}}-2log\pi_{2}\right), \end{array}$$

and in Class 2 otherwise. In Eq. (3) *s*_0 _is the median value of the *s_g _*over the set of variables, and ${\bar{x}}_{g}^{,(1)}$ and ${\bar{x}}_{g}^{,(2)}$ are Class 1 and Class 2 shrunken centroids for variable *g*, respectively. The second term in the equation is a correction based on the class prior probability *π_k_*.

We used the functions pamr.train and pamr.predict from the *pamr *package. The threshold value in each step of the simulation was set to the largest threshold with the smallest number of misclassification error in the training set.

#### PLR

Logistic regression cannot be used when the number of variables exceeds the number of observations. Penalized logistic regression (PLR, [42]) avoids this problem by considering a penalized log likelihood, which for a two class problem and a quadratic penalty is given by

(4) $$\begin{array}{c} l(\alpha ,\beta )=-\sum_{i=1}^{n}(y_{i}\log p_{i}+(1-y_{i})\log(1-p_{i}))+\\ +\frac{\lambda }{2}\sum_{g=1}^{G}\beta_{g}^{2}, \end{array}$$

where *y_i _*denotes the response for *i*th individual (1 for Class 1 and 0 for Class 2), and *p_i _*is the probability of belonging to Class 1 for a sample with variables **x**_*i *_(*p_i _*= *P *(*y_i _*= 1|**x**_*i*_)), which is a function of the observed data **x **and of the regression coefficients (*α *and *β*). The penalization parameter *λ *can be chosen or estimated (by cross validation or using Akaike's information criterion), while *α *and *β *are estimated (using Newton-Raphson procedure). For a new sample with features x* the probabilities to belong to Class 1 or Class 2 are estimated ($\hat{p}$ and 1 -$\hat{p}$) and the sample is classified in Class 1 if $\hat{p}$ > 1 -$\hat{p}$, i.e., if $\hat{p}$ > 0.5, and in Class 2 otherwise, with ties broken at random.

Another possibility is to classify a sample in Class 1 if $\hat{p}>k_{1}^{train}$; we refer to the results obtained using this classification rule as those based on the theoretical threshold (PLR-THR).

We used the functions plr and predict.plr from the *stepPlr *package. We used *λ *= 1 for all the analyses; this choice was determined after an exploratory analysis that showed that in our simulation settings using any value for *λ *in the range from 0 to 1.5 had little effect on the classification rules. However, we observed that large values of *λ *worsened the performance of PLR when the training set was highly imbalanced.

### Data simulation

We simulated *p *= 40, 1000 or 10000 independent variables for each of *n *= 100 samples. Under the null case all the variables were simulated independently from the standard normal distribution (mean *μ*= 0 and standard deviation *σ *= 1, *N*(0, 1)) and the class membership of the samples was randomly assigned. Under the alternative case, class membership was dependent on variables; for each sample, *p*_0 _variables were generated independently from *N*(0, 1) (null-variables), while the remaining variables (*p_DE_*, non-null variables) were generated independently from a normal distribution with mean *μ*^(2) ^and standard deviation *σ *= 1 for samples from Class 2, and from *N*(0, 1) for samples from Class 1. Different values of *μ*^(2) ^(*μ*^(2) ^= 0.1, 0.2,...,1.9, 2,3) and of *p_DE _*(*p_DE _*= 20, 40 or *p*) were considered.

The data set was split into a training set (*n_train _*= 80 samples) and a test set (*n_test _*= 20 samples). Different levels of imbalance between the two classes were considered, varying the proportion of samples from Class 1 from 5% to 95% (*k*_1 _= 0.05, 0.10,..., 0.95). We looked at situations where we had (i) imbalanced training sets ($k_{1}^{train}$ = 0.05, ..., 0.95) and balanced test sets ($k_{1}^{test}$ = 0.50), (ii) the same imbalance in training and test set ($k_{1}^{train}$ = $k_{1}^{test}$ = 0.05, ..., 0.95) and (iii) balanced training set and imbalanced test set ($k_{1}^{train}$ = 0.50, $k_{1}^{test}$ = 0.05, ..., 0.95). In a limited set of simulations we also used smaller sample size (*n *= 70, *n_train _*= 50, *n_test _*= 20). All the simulations were repeated 1,000 times.

### Derivation of the classification rules

#### Normalization

We evaluated the effect of data normalization, developing classification rules (i) without normalizing data (i.e., using raw data *x_ij_*), (ii) normalizing the samples (i.e., setting the mean expression for each sample equal to zero, using $x_{ij}^{s}=x_{ij}-\frac{1}{p}\sum_{k=1}^{p}x_{ik}$ and (iii) normalizing the variables (i.e., setting the mean expression for each variable equal to zero, using.$x_{ij}^{v}=x_{ij}-\frac{1}{n}\sum_{k=1}^{n}x_{kj}$. Normalizations were performed separately on training and test set.

#### Variable selection

Variable selection was performed exclusively on the training set, selecting the *G *genes with the largest absolute value of the univariate two-sample t test statistic (*G *= 40); we also considered the situation where all the variables were used (*G *= *p*). The classification rules were derived completely on the training set, using the variables selected on the training set and the six classification methods described in the section Classification methods.

### Evaluation of the performance of the classifiers

The performance of the classifiers was evaluated on the test set. It is well know that for imbalanced data the proportion of correctly classified samples can be a misleading measure of the performance of a classifier ([29], chapter 2). For this reason three different measures of accuracy were considered: (i) overall predictive accuracy (PA, the number of correctly classified samples from the test set divided by the total number of samples in the test set), (ii) predictive accuracy of Class 1 (PA_1_=*P *(Predict Class_1_|True Class_1_), i.e., *PA *evaluated using only samples from Class 1), (iii) predictive accuracy of Class 2 (PA_2_).

Predictive accuracy can be seen as the weighted average of the class specific predictive accuracies, with weights equal to the proportion of samples each class:

(5) $$\text{PA}={\text{PA}}_{1}*k_{1}^{test}+{\text{PA}}_{2}*k_{2}^{test}.$$

We derived the 95% prediction intervals for the overall PA as the 2.5th percentile to the 97.5th percentile from the distribution of PAs obtained from 1000 replications, as described in [43].

We evaluated also the predictive values for both classes, i.e., the probabilities of predicting the true class membership: PV_*i *_= *P *(True Class_*i*_|Predict Class_*i*_). The predictive values depend on the proportion of samples from each class in the population (*κ_i _*is the proportion of samples from Class *i *in the population).

(6) $$\begin{array}{l} {\text{PV}}_{1}=\frac{\kappa_{1}{\text{PA}}_{1}}{\kappa_{1}{\text{PA}}_{1}+(1-\kappa_{1})(1-{\text{PA}}_{2})},\\ {\text{PV}}_{2}=\frac{(1-\kappa_{1}){\text{PA}}_{2}}{\left(1-\kappa_{1}){\text{PA}}_{2}+\kappa_{1}(1-{\text{PA}}_{1}\right)}. \end{array}$$

If not otherwise stated, we assumed that the proportion of samples in each class was the same in the training set and in the population ($\kappa_{i}=k_{i}^{train}$).

In biomedical research the two classes often refer to a disease status (positive or negative), and sensitivity (true positive fraction) and specificity (1-false positive fraction) are used to describe the accuracy of the classifier. Assuming that Class 1 is the positive class, PA_1 _is the sensitivity of the classifier and PA_2 _is its specificity. Furthermore, PV_1 _is the positive predictive value (PPV) and PV_2 _is the negative predictive value (NPV).

We calculated also the area under the receiver operating characteristic (ROC) curve (AUC) ([29], chapter 4), using the *hmisc *package.

### Solutions for the development of classifiers for class-imbalanced data

#### Over-sampling

Simple over-sampling consists in obtaining a class-balanced training set replicating a subset of randomly selected samples from the minority class (replicating *max*(*n*_1_, *n*_2_) - *min*(*n*_1_, *n*_2_) samples from the minority class and obtaining a training set of size 2*max*(*n*_1_, *n*_2_)) [19,44,45]. The classification rule was derived on the resampled training set as described for the original data and evaluated on the test set. The classification rule is derived on the replicated training set as described for the original data and evaluated on the test set.

#### Down-sizing

Simple down-sizing consists in obtaining a class-balanced training set by removing a subset of randomly selected samples from the majority class (removing *max*(*n*_1_, *n*_2_) - *min*(*n*_1_, *n*_2_) samples from the majority class, obtaining a training set of size 2*min*(*n*_1_, *n*_2_)) [19,45]. The classification rule is derived on the reduced training set as described for the original data and evaluated on the test set.

#### Multiple down-sizing (MultDS, asymmetric bagging with embedded variable selection)

With multiple down-sizing (MultDS) we tried to make use of the whole information available in the majority class by performing down-sizing multiple times. 101 random selections of samples from the majority class were made and classification rule was derived on each of the 101 balanced training sets (note that the minority class was the same in each training set). The 101 down-sized classifiers were combined by majority voting: the class assignments for new samples were obtained from each of the down-sized classifier and the new samples were assigned to the class with the larger number of votes. Multiple down-sizing can be seen as a special case of asymmetric bagging [24], except that the variables are selected in each down-sized training set.

#### Microarray Data

Sotiriou et al. [28] analyzed cDNA gene expression profiles from 99 tumor specimens from breast cancer patients. In addition to gene expression values for 7650 genes (probes) preprocessed as described in Sotiriou et al. (2003), there was standard prognostic variable information available for each patient (the data are publicly available at http://linus.nci.nih.gov/~brb/DataArchive.html). Missing log-expression values were replaced with 0. Here we considered two two-class prediction problems: the first was to predict estrogen receptor (ER) status, which was negative (ER-) for 34 patients and positive (ER+) for 65 patients, according to ligand-binding assay; the second was to predict the grade of tumors, which was 1 or 2 for 54 patients and 3 for 45 patients.

On these data we evaluated the performance of all the classifiers used in the simulation studies, obtaining different levels of class imbalance in the training and test sets by including a selected subset of the samples in the analyses. For each setting (imbalance level) we replicated the analysis 500 times, by randomly selecting which samples to include in the training and in the test set. Variable selection consisted in selecting on each training set the 40 probes with the largest absolute value of the univariate statistic derived from the two-sided t test with equal variances. Overall PA, class specific PA and PV, and AUC were obtained averaging the results obtained from the 500 analyses. We evaluated the classifiers both without applying any specific corrections for class imbalance, and using the solutions presented in the previous paragraph for the simulation studies (over-sampling, down-sizing and multiple down-sizing). We evaluated the 95% prediction intervals for the class specific PAs using 500 replications.

## Abbreviations

AUC: area under the ROC curve; DLDA: diagonal linear discriminant analysis; DQDA: diagonal quadratic discriminant analysis; ER: estrogen receptor; ER+: positive estrogen receptor status; ER-: negative estrogen receptor status; k-NN: nearest neighbor classifier with *k *neighbors; LOOCV: leave-one-out cross-validation; MultDS: multiple down-sizing; PA: predictive accuracy; PA_1_: predictive accuracy for Class 1; PA_2_: predictive accuracy for Class 2; PAM: prediction analysis of microarrays; PLR: penalized logistic regression; PLR-THR: penalized logistic regression with adjusted threshold; PV: predictive value; PV_1_: predictive value for Class 1; PV_2_: predictive value for Class 2; RF: random forests; RF-THR: random forests with adjusted threshold; SVM: support vector machines

## Authors' contributions

RB performed the computations and wrote the manuscript; LL designed research and wrote the manuscript. Both authors read and approved the final manuscript.

## Supplementary Material

Additional file 1 **Behavior of the classifiers under the null hypothesis, using test sets that are balanced and have different sample size**. The additional file reports in a table format the same results presented graphically in Figure 1; here the results refer to a balanced test set and compare two different sample sizes of test set. Besides predictive accuracies, also predictive values and AUC are reported. Click here for file

Additional file 2 **Behavior of eight variable selection methods under the null and alternative hypothesis**. Click here for file

Additional file 3 **Predictive accuracies, predictive values and area under the ROC curve for nine classifiers under the alternative hypothesis**. The additional file presents in a table format the complete simulation results shown grafically in Figure 5, first column. Predictive accuaracies, predictive values and AUC are reported. Click here for file

Additional file 4 **Effect of performing variable selection and increasing the number of variables for the nine classifiers**. The additional file reports the same results described in the left panels of Figure 3 for 1-NN, DLDA and PLR, but for all the classifiers. Click here for file

Additional file 5 **Effect of performing variable selection and increasing the number of variables when all the variables are different between the two classes, for the nine classifiers**. The additional file reports the same results described in the right panels of Figure 3 for 1-NN, DLDA and PLR, but for all the classifiers. Click here for file

Additional file 6 **Effect of not performing variable selection**. The additional file reports the predictive accuracy results obtained for the nine classifiers, when the number of variables is large (*p *= 1000) and variable selection is not performed. The simulation setting is the same as for Figure 5, first column. Click here for file

Additional file 7 **Effect of varying the magnitude of the difference between classes**. The additional file shows, for the nine classifiers, the same results presented in Figure 4 for 1-NN, DLDA and PLR. Click here for file

Additional file 8 **Solutions to the class imbalance problem**. The additional file shows, for the nine classifiers, the same results presented in Figure 5 for 3-NN, DLDA and PLR. Click here for file

Additional file 9 **Over-sampling and variable centering**. The additional file shows the behavior of the classifiers using over-sampling and mean-centering the variables. An explanation for the improved behavior of DLDA is given. Click here for file

Additional file 10 **Variability of the overall predictive accuracy**. The figure shows, for four classifiers, the overall predictive accuracy and its 95% prediction intervals (obtained with no correction, over-sampling, down-sizing and multiple down-sizing). The simulation setting is the same as described for Figure 5, but the test set contained 500 samples. Click here for file

Additional file 11 **Threshold based on class imbalance for classification with PLR and RF**. The figure shows, for RF and PLR, the overall (PA) and class specific predictive accuracy (PA_1 _and PA_2_) obtained in the same simulation setting of Figure 5: here the classification rule was based on the threshold equal to the class imbalance of the training set (RF-THR and PLR-THR). Click here for file

Additional file 12 **Overall predictive accuracy and the 95% prediction intervals for the prediction of ER status**. The figure shows for all the classifiers the same results as those presented in Figure 6. Click here for file

Additional file 13 **Behavior of the nine classifiers for the prediction of ER status - no correction**. The table shows, for all the classifiers, the predictive accuracies presented in Figure 6 (no correction for class imbalance), together with the predictive values and AUC. Click here for file

Additional file 14 **Behavior of the nine classifiers for the prediction of ER status using multiple down-sizing**. The table shows, for all the classifiers, the predictive accuracies obtained in Figure 6 using multiple down-sizing, together with the predictive values and AUC. Click here for file
