# Supplementary material for: Class prediction for high-dimensional class-imbalanced data
Source: BMC Bioinformatics. 2010 Oct 20;11:523. doi: 10.1186/1471-2105-11-523 (PMC3098087; doi:10.1186/1471-2105-11-523)
Supplement: Additional file 1 — Behavior of the classifiers under the null hypothesis, using test sets that are balanced and have different sample size. The additional file reports in a table format the same results presented graphically in Figure 1; here the results refer to a balanced test set and compare two different sample sizes of test set. Besides predictive accuracies, also predictive values and AUC are reported. [file 1471-2105-11-523-S1.PDF]

1-NN

k_1^{train}	n_{test}	PA	PA_1	PA_2	PV_1	PV_2	AUC
0.1	20	0.5	0.05	0.95	NA	0.9	0.5
		(0.046)	(0.068)	(0.069)	(NA)	(0.009)	(0.109)
	500	0.5	0.05	0.95	0.11	0.9	0.5
		(0.009)	(0.019)	(0.018)	(0.046)	(0.002)	(0.022)
0.2	20	0.5	0.15	0.84	0.32	0.8	0.49
		(0.084)	(0.118)	(0.12)	(0.335)	(0.035)	(0.111)
	500	0.5	0.16	0.84	0.2	0.8	0.5
		(0.016)	(0.037)	(0.038)	(0.035)	(0.006)	(0.021)
0.3	20	0.5	0.27	0.73	0.34	0.7	0.5
		(0.102)	(0.153)	(0.148)	(0.222)	(0.064)	(0.105)
	500	0.5	0.27	0.73	0.3	0.7	0.5
		(0.019)	(0.048)	(0.049)	(0.031)	(0.011)	(0.022)
0.4	20	0.5	0.38	0.62	0.41	0.6	0.5
		(0.109)	(0.162)	(0.158)	(0.156)	(0.093)	(0.11)
	500	0.5	0.38	0.61	0.4	0.6	0.5
		(0.021)	(0.053)	(0.055)	(0.027)	(0.017)	(0.022)
0.5	20	0.5	0.5	0.5	0.5	0.5	0.5
		(0.116)	(0.164)	(0.17)	(0.127)	(0.126)	(0.112)
	500	0.5	0.5	0.5	0.5	0.5	0.5
		(0.023)	(0.056)	(0.057)	(0.023)	(0.023)	(0.023)

3-NN

k_1^{train}	n_{test}	PA	PA_1	PA_2	PV_1	PV_2	AUC
0.1	20	0.5	0.05	0.95	NA	0.9	0.5
		(0.046)	(0.068)	(0.069)	(NA)	(0.009)	(0.162)
	500	0.5	0.05	0.95	0.11	0.9	0.5
		(0.009)	(0.019)	(0.018)	(0.046)	(0.002)	(0.033)
0.2	20	0.5	0.15	0.84	0.32	0.8	0.5
		(0.084)	(0.118)	(0.12)	(0.335)	(0.035)	(0.144)
	500	0.5	0.16	0.84	0.2	0.8	0.5
		(0.016)	(0.037)	(0.038)	(0.035)	(0.006)	(0.028)
0.3	20	0.5	0.27	0.73	0.34	0.7	0.5
		(0.102)	(0.153)	(0.148)	(0.222)	(0.064)	(0.129)
	500	0.5	0.27	0.73	0.3	0.7	0.5
		(0.019)	(0.048)	(0.049)	(0.031)	(0.011)	(0.026)
0.4	20	0.5	0.38	0.62	0.41	0.6	0.5
		(0.109)	(0.162)	(0.158)	(0.156)	(0.093)	(0.12)
	500	0.5	0.38	0.61	0.4	0.6	0.5
		(0.021)	(0.053)	(0.055)	(0.027)	(0.017)	(0.025)
0.5	20	0.5	0.5	0.5	0.5	0.5	0.5
		(0.116)	(0.164)	(0.17)	(0.127)	(0.126)	(0.125)
	500	0.5	0.5	0.5	0.5	0.5	0.5
		(0.023)	(0.056)	(0.057)	(0.023)	(0.023)	(0.024)

5-NN

k_1^{train}	n_{test}	PA	PA_1	PA_2	PV_1	PV_2	AUC
0.1	20	0.5	0.05	0.95	NA	0.9	0.5
		(0.046)	(0.068)	(0.069)	(NA)	(0.009)	(0.184)
	500	0.5	0.05	0.95	0.11	0.9	0.5
		(0.009)	(0.019)	(0.018)	(0.046)	(0.002)	(0.038)
0.2	20	0.5	0.15	0.84	0.32	0.8	0.5
		(0.084)	(0.118)	(0.12)	(0.335)	(0.035)	(0.149)
	500	0.5	0.16	0.84	0.2	0.8	0.5
		(0.016)	(0.037)	(0.038)	(0.035)	(0.006)	(0.03)
0.3	20	0.5	0.27	0.73	0.34	0.7	0.5
		(0.102)	(0.153)	(0.148)	(0.222)	(0.064)	(0.138)
	500	0.5	0.27	0.73	0.3	0.7	0.5
		(0.019)	(0.048)	(0.049)	(0.031)	(0.011)	(0.026)
0.4	20	0.5	0.38	0.62	0.41	0.6	0.5
		(0.109)	(0.162)	(0.158)	(0.156)	(0.093)	(0.123)
	500	0.5	0.38	0.61	0.4	0.6	0.5
		(0.021)	(0.053)	(0.055)	(0.027)	(0.017)	(0.025)
0.5	20	0.5	0.5	0.5	0.5	0.5	0.5
		(0.116)	(0.164)	(0.17)	(0.127)	(0.126)	(0.126)
	500	0.5	0.5	0.5	0.5	0.5	0.5
		(0.023)	(0.056)	(0.057)	(0.023)	(0.023)	(0.025)

DLDA

k_1^{train}	n_{test}	PA	PA_1	PA_2	PV_1	PV_2	AUC
0.1	20	0.5	0.05	0.95	NA	0.9	0.49
		(0.046)	(0.068)	(0.069)	(NA)	(0.009)	(0.222)
	500	0.5	0.05	0.95	0.11	0.9	0.5
		(0.009)	(0.019)	(0.018)	(0.046)	(0.002)	(0.042)
0.2	20	0.5	0.15	0.84	0.32	0.8	0.49
		(0.084)	(0.118)	(0.12)	(0.335)	(0.035)	(0.163)
	500	0.5	0.16	0.84	0.2	0.8	0.5
		(0.016)	(0.037)	(0.038)	(0.035)	(0.006)	(0.032)
0.3	20	0.5	0.27	0.73	0.34	0.7	0.5
		(0.102)	(0.153)	(0.148)	(0.222)	(0.064)	(0.144)
	500	0.5	0.27	0.73	0.3	0.7	0.5
		(0.019)	(0.048)	(0.049)	(0.031)	(0.011)	(0.028)
0.4	20	0.5	0.38	0.62	0.41	0.6	0.5
		(0.109)	(0.162)	(0.158)	(0.156)	(0.093)	(0.135)
	500	0.5	0.38	0.61	0.4	0.6	0.5
		(0.021)	(0.053)	(0.055)	(0.027)	(0.017)	(0.026)
0.5	20	0.5	0.5	0.5	0.5	0.5	0.5
		(0.116)	(0.164)	(0.17)	(0.127)	(0.126)	(0.133)
	500	0.5	0.5	0.5	0.5	0.5	0.5
		(0.023)	(0.056)	(0.057)	(0.023)	(0.023)	(0.027)

DQDA

k_1^{train}	n_{test}	PA	PA_1	PA_2	PV_1	PV_2	AUC
0.1	20	0.5	0.05	0.95	NA	0.9	0.49
		(0.046)	(0.068)	(0.069)	(NA)	(0.009)	(0.222)
	500	0.5	0.05	0.95	0.11	0.9	0.5
		(0.009)	(0.019)	(0.018)	(0.046)	(0.002)	(0.042)
0.2	20	0.5	0.15	0.84	0.32	0.8	0.49
		(0.084)	(0.118)	(0.12)	(0.335)	(0.035)	(0.166)
	500	0.5	0.16	0.84	0.2	0.8	0.5
		(0.016)	(0.037)	(0.038)	(0.035)	(0.006)	(0.033)
0.3	20	0.5	0.27	0.73	0.34	0.7	0.5
		(0.102)	(0.153)	(0.148)	(0.222)	(0.064)	(0.141)
	500	0.5	0.27	0.73	0.3	0.7	0.5
		(0.019)	(0.048)	(0.049)	(0.031)	(0.011)	(0.029)
0.4	20	0.5	0.38	0.62	0.41	0.6	0.51
		(0.109)	(0.162)	(0.158)	(0.156)	(0.093)	(0.135)
	500	0.5	0.38	0.61	0.4	0.6	0.5
		(0.021)	(0.053)	(0.055)	(0.027)	(0.017)	(0.026)
0.5	20	0.5	0.5	0.5	0.5	0.5	0.5
		(0.116)	(0.164)	(0.17)	(0.127)	(0.126)	(0.132)
	500	0.5	0.5	0.5	0.5	0.5	0.5
		(0.023)	(0.056)	(0.057)	(0.023)	(0.023)	(0.027)

RF

k_1^{train}	n_{test}	PA	PA_1	PA_2	PV_1	PV_2	AUC
0.1	20	0.5	0.05	0.95	NA	0.9	0.49
		(0.046)	(0.068)	(0.069)	(NA)	(0.009)	(0.217)
	500	0.5	0.05	0.95	0.11	0.9	0.5
		(0.009)	(0.019)	(0.018)	(0.046)	(0.002)	(0.042)
0.2	20	0.5	0.15	0.84	0.32	0.8	0.49
		(0.084)	(0.118)	(0.12)	(0.335)	(0.035)	(0.163)
	500	0.5	0.16	0.84	0.2	0.8	0.5
		(0.016)	(0.037)	(0.038)	(0.035)	(0.006)	(0.033)
0.3	20	0.5	0.27	0.73	0.34	0.7	0.5
		(0.102)	(0.153)	(0.148)	(0.222)	(0.064)	(0.137)
	500	0.5	0.27	0.73	0.3	0.7	0.5
		(0.019)	(0.048)	(0.049)	(0.031)	(0.011)	(0.028)
0.4	20	0.5	0.38	0.62	0.41	0.6	0.5
		(0.109)	(0.162)	(0.158)	(0.156)	(0.093)	(0.134)
	500	0.5	0.38	0.61	0.4	0.6	0.5
		(0.021)	(0.053)	(0.055)	(0.027)	(0.017)	(0.026)
0.5	20	0.5	0.5	0.5	0.5	0.5	0.5
		(0.116)	(0.164)	(0.17)	(0.127)	(0.126)	(0.135)
	500	0.5	0.5	0.5	0.5	0.5	0.5
		(0.023)	(0.056)	(0.057)	(0.023)	(0.023)	(0.026)

SVM

k_1^{train}	n_{test}	PA	PA_1	PA_2	PV_1	PV_2	AUC
0.1	20	0.5	0.05	0.95	NA	0.9	0.49
		(0.046)	(0.068)	(0.069)	(NA)	(0.009)	(0.225)
	500	0.5	0.05	0.95	0.11	0.9	0.5
		(0.009)	(0.019)	(0.018)	(0.046)	(0.002)	(0.042)
0.2	20	0.5	0.15	0.84	0.32	0.8	0.5
		(0.084)	(0.118)	(0.12)	(0.335)	(0.035)	(0.164)
	500	0.5	0.16	0.84	0.2	0.8	0.5
		(0.016)	(0.037)	(0.038)	(0.035)	(0.006)	(0.032)
0.3	20	0.5	0.27	0.73	0.34	0.7	0.5
		(0.102)	(0.153)	(0.148)	(0.222)	(0.064)	(0.144)
	500	0.5	0.27	0.73	0.3	0.7	0.5
		(0.019)	(0.048)	(0.049)	(0.031)	(0.011)	(0.028)
0.4	20	0.5	0.38	0.62	0.41	0.6	0.5
		(0.109)	(0.162)	(0.158)	(0.156)	(0.093)	(0.137)
	500	0.5	0.38	0.61	0.4	0.6	0.5
		(0.021)	(0.053)	(0.055)	(0.027)	(0.017)	(0.026)
0.5	20	0.5	0.5	0.5	0.5	0.5	0.5
		(0.116)	(0.164)	(0.17)	(0.127)	(0.126)	(0.13)
	500	0.5	0.5	0.5	0.5	0.5	0.5
		(0.023)	(0.056)	(0.057)	(0.023)	(0.023)	(0.026)

PAM

k_1^{train}	n_{test}	PA	PA_1	PA_2	PV_1	PV_2	AUC
0.1	20	0.5	0.05	0.95	NA	0.9	0.49
		(0.046)	(0.068)	(0.069)	(NA)	(0.009)	(0.223)
	500	0.5	0.05	0.95	0.11	0.9	0.5
		(0.009)	(0.019)	(0.018)	(0.046)	(0.002)	(0.042)
0.2	20	0.5	0.15	0.84	0.32	0.8	0.49
		(0.084)	(0.118)	(0.12)	(0.335)	(0.035)	(0.163)
	500	0.5	0.16	0.84	0.2	0.8	0.5
		(0.016)	(0.037)	(0.038)	(0.035)	(0.006)	(0.032)
0.3	20	0.5	0.27	0.73	0.34	0.7	0.5
		(0.102)	(0.153)	(0.148)	(0.222)	(0.064)	(0.144)
	500	0.5	0.27	0.73	0.3	0.7	0.5
		(0.019)	(0.048)	(0.049)	(0.031)	(0.011)	(0.028)
0.4	20	0.5	0.38	0.62	0.41	0.6	0.5
		(0.109)	(0.162)	(0.158)	(0.156)	(0.093)	(0.135)
	500	0.5	0.38	0.61	0.4	0.6	0.5
		(0.021)	(0.053)	(0.055)	(0.027)	(0.017)	(0.026)
0.5	20	0.5	0.5	0.5	0.5	0.5	0.5
		(0.116)	(0.164)	(0.17)	(0.127)	(0.126)	(0.133)
	500	0.5	0.5	0.5	0.5	0.5	0.5
		(0.023)	(0.056)	(0.057)	(0.023)	(0.023)	(0.027)

PLR

k_1^{train}	n_{test}	PA	PA_1	PA_2	PV_1	PV_2	AUC
0.1	20	0.5	0.05	0.95	NA	0.9	0.5
		(0.046)	(0.068)	(0.069)	(NA)	(0.009)	(0.226)
	500	0.5	0.05	0.95	0.11	0.9	0.5
		(0.009)	(0.019)	(0.018)	(0.046)	(0.002)	(0.043)
0.2	20	0.5	0.15	0.84	0.32	0.8	0.49
		(0.084)	(0.118)	(0.12)	(0.335)	(0.035)	(0.162)
	500	0.5	0.16	0.84	0.2	0.8	0.5
		(0.016)	(0.037)	(0.038)	(0.035)	(0.006)	(0.032)
0.3	20	0.5	0.27	0.73	0.34	0.7	0.5
		(0.102)	(0.153)	(0.148)	(0.222)	(0.064)	(0.146)
	500	0.5	0.27	0.73	0.3	0.7	0.5
		(0.019)	(0.048)	(0.049)	(0.031)	(0.011)	(0.028)
0.4	20	0.5	0.38	0.62	0.41	0.6	0.5
		(0.109)	(0.162)	(0.158)	(0.156)	(0.093)	(0.134)
	500	0.5	0.38	0.61	0.4	0.6	0.5
		(0.021)	(0.053)	(0.055)	(0.027)	(0.017)	(0.026)
0.5	20	0.5	0.5	0.5	0.5	0.5	0.5
		(0.116)	(0.164)	(0.17)	(0.127)	(0.126)	(0.133)
	500	0.5	0.5	0.5	0.5	0.5	0.5
		(0.023)	(0.056)	(0.057)	(0.023)	(0.023)	(0.026)

Table 1: Predictive accuracy (PA), class 1 PA (PA_1), class 2 PA (PA_2), positive and negative predictive value (PV_1, PV_2) for different proportions of class 1 samples in the training set (k_1^{train}, $n_{train} = 80$). Test set contained 20 or 500 samples (n_{test}) and proportion of class 1 samples was the same as in the training set. 40 variables were simulated from $N(0,1)$ distribution for both classes and all were used for derivation of classification rule.
