# Supplementary material for: Class prediction for high-dimensional class-imbalanced data
Source: BMC Bioinformatics. 2010 Oct 20;11:523. doi: 10.1186/1471-2105-11-523 (PMC3098087; doi:10.1186/1471-2105-11-523)
Supplement: Additional file 2 — Behavior of eight variable selection methods under the null and alternative hypothesis. [file 1471-2105-11-523-S2.PDF]

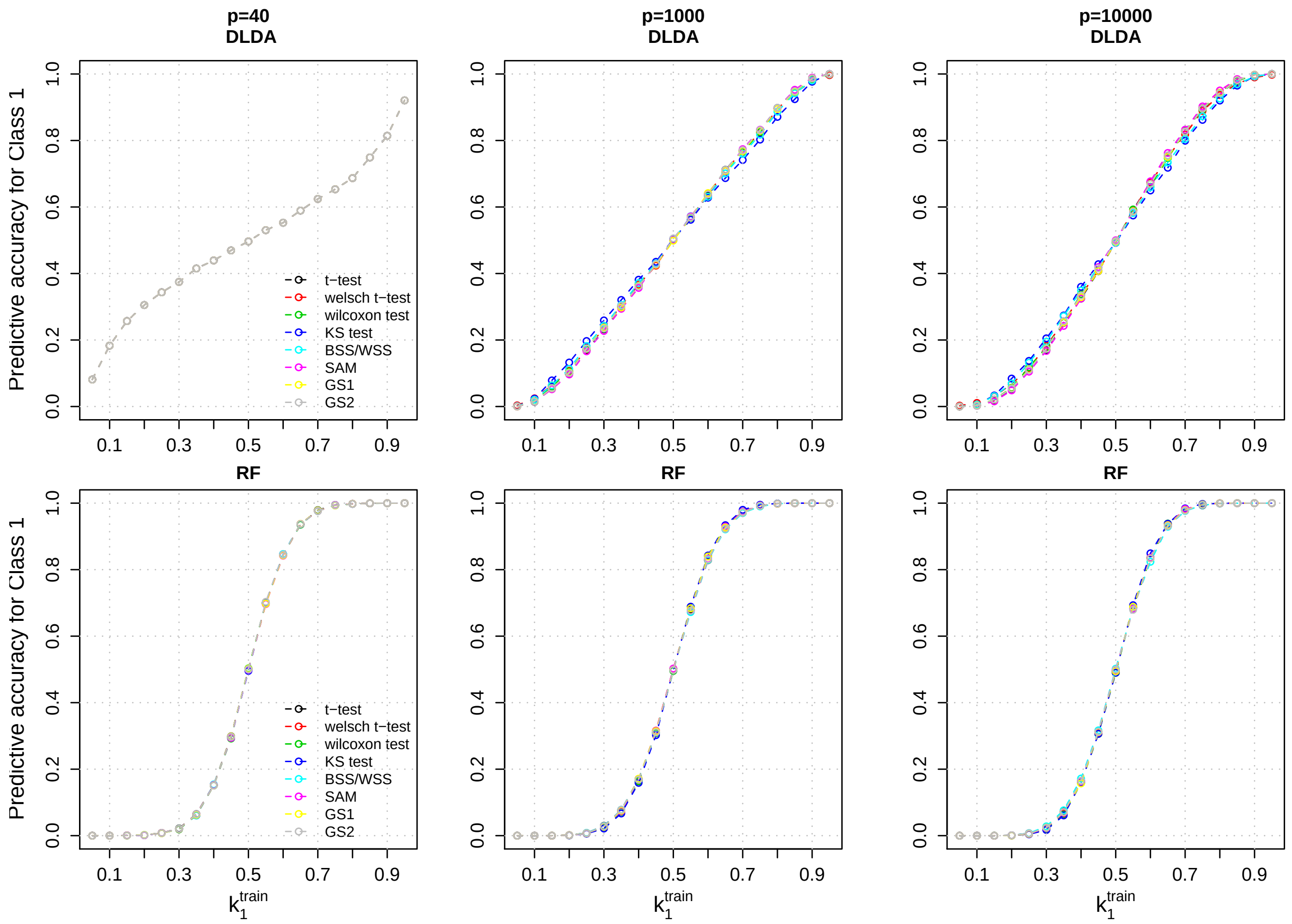

Figure 1: Null case. Class 1 predictive accuracy (PA_1) varying the proportion of samples from Class 1 in the training set (k_1^{train}); the test set contained 80 samples and $p = 40, 1000$ or 10000 variables were generated from $N(0, 1)$ for both classes. Eight different variable selection methods were used to select the $G = 40$ variables that were included in classification (see legend for more details). The test set contained an equal number of Class 1 and Class 2 samples (10 from each class). Samples were mean centered. Details on data generation are reported in the Methods section.

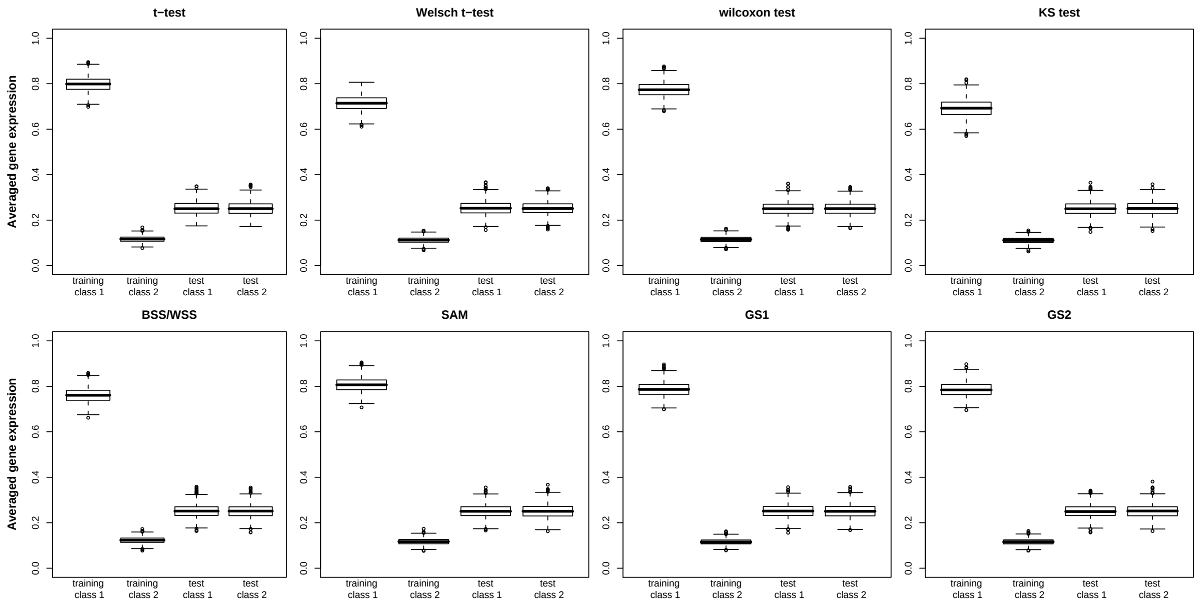

Figure 2: Null case. Box and whisker plots of the absolute values of the sample means of the $G = 40$ selected variables, in the training and in the test set. 10000 variables from $N(0, 1)$ were generated for 8 Class 1 samples and for 72 Class 2 samples. Each plot shows the results obtained using a different variable selection method.

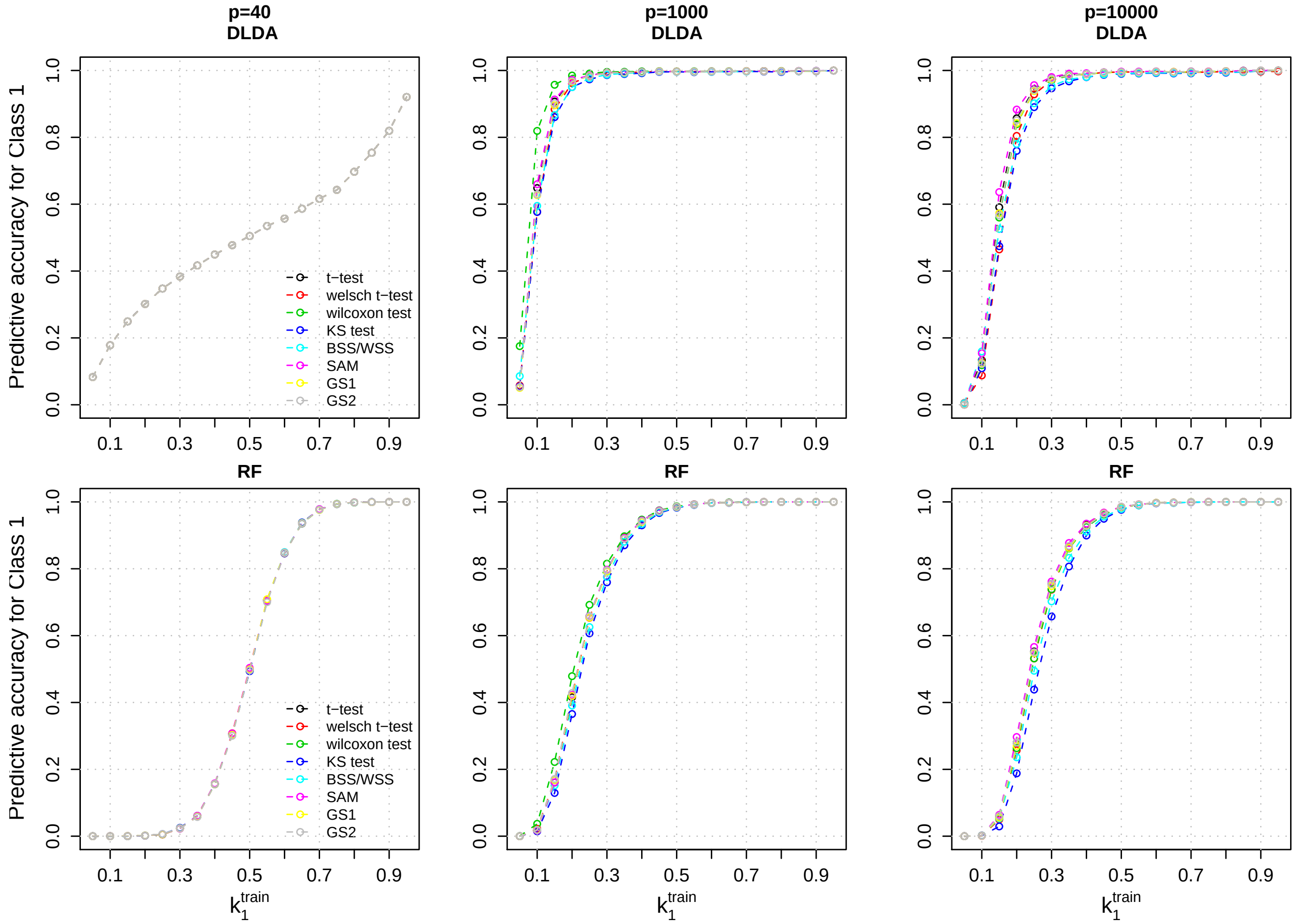

Figure 3: Alternative case. Class 1 predictive accuracy (PA_1) varying the proportion of Class 1 samples in the training set (k_1^{train}); the test set contained 80 samples. $p = 40, 1000$ or 10000 variables were generated. For Class 1 all the variables were simulated from the same distribution ($N(0,1)$), while for Class 2 $p_{DE} = 40$ variables were generated from $N(1,1)$, and the other $p - p_{DE}$ variables were generated from $N(0,1)$. $G = 40$ variables were selected using different variable selection methods. The test set contained 20 samples and was balanced. Samples were mean centered. Details on data generation are reported in the Methods section.

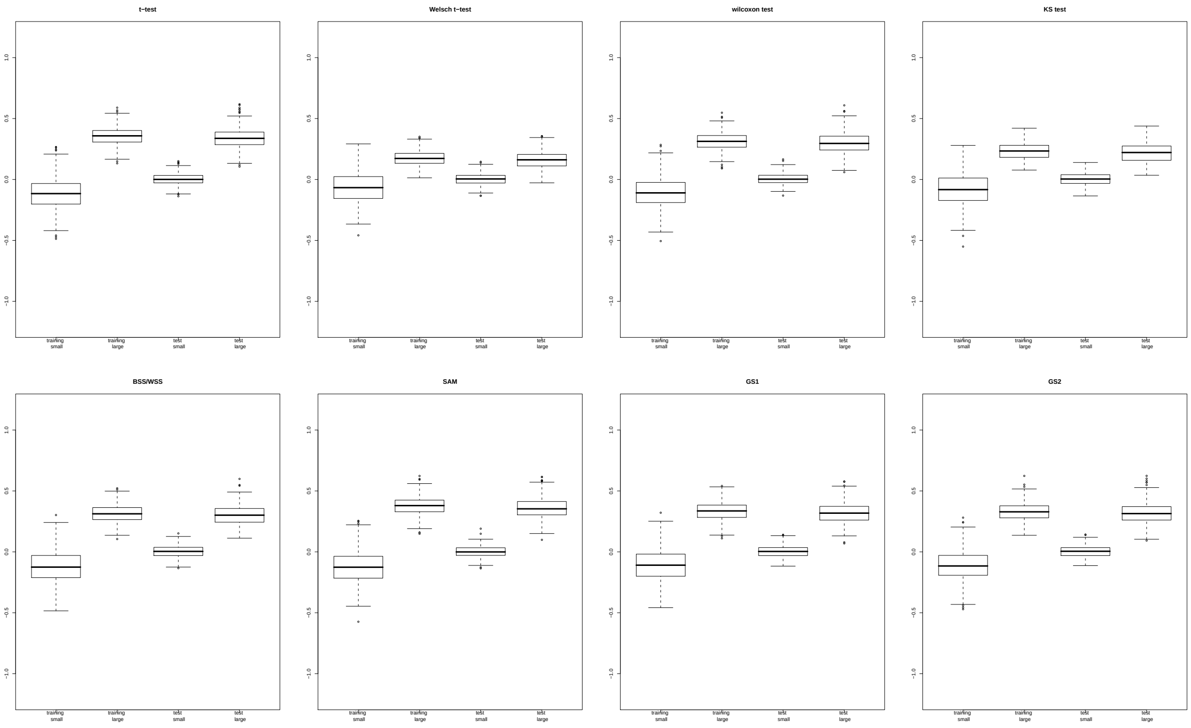

Figure 4: Alternative case. Box and whisker plots of the absolute values of the sample means of the $G = 40$ selected variables, in the training and in the test set. $10,000$ variables were simulated, the simulation setting is the same as described in Figure 3 of this file.

Legend

- t-test: t-test with equal variances assumed
- welsch t-test: t-test for different between class variances
- wilcoxon test: wilcoxon non-parametric test for difference between two independent groups
- KS test: Kolmogorov-Smirnov non-parametric test of equality of one-dimensional probability distributions
- BSS/WSS: ratio of between to within subject variability (Dudoit et al., JASA, pp. 77-87, 2002)
- SAM: significance analysis of microarrays (Tusher et al., PNAS, pp. 5116-5121, 2001)
- GS1, GS2 (Yang et al., BMC Bioinformatics, p. 228, 2006)
