# Supplementary material for: Class prediction for high-dimensional class-imbalanced data
Source: BMC Bioinformatics. 2010 Oct 20;11:523. doi: 10.1186/1471-2105-11-523 (PMC3098087; doi:10.1186/1471-2105-11-523)
Supplement: Additional file 3 — Predictive accuracies, predictive values and area under the ROC curve for nine classifiers under the alternative hypothesis. The additional file presents in a table format the complete simulation results shown grafically in Figure 5, first column. Predictive accuaracies, predictive values and AUC are reported. [file 1471-2105-11-523-S3.PDF]

k_1^{train}	n_{test}	1-NN PA	1-NN PA_1	1-NN PA_2	1-NN PV_1	1-NN PV_2	1-NN AUC	3-NN PA	3-NN PA_1	3-NN PA_2	3-NN PV_1	3-NN PV_2	3-NN AUC	5-NN PA	5-NN PA_1	5-NN PA_2	5-NN PV_1	5-NN PV_2	5-NN AUC
0.1	20	0.57	0.14	1	NA	0.91	0.57	0.54	0.08	1	NA	0.91	0.64	0.52	0.05	1	NA	0.9	0.68
		(0.059)	(0.118)	(0.021)	(NA)	(0.011)	(0.067)	(0.046)	(0.092)	(0.008)	(NA)	(0.009)	(0.093)	(0.036)	(0.072)	(0.003)	(NA)	(0.007)	(0.098)
	500	0.57	0.13	1	0.86	0.91	0.57	0.54	0.08	1	0.96	0.91	0.64	0.52	0.05	1	0.99	0.9	0.68
		(0.036)	(0.073)	(0.005)	(0.177)	(0.007)	(0.034)	(0.026)	(0.052)	(0.002)	(0.128)	(0.005)	(0.053)	(0.02)	(0.039)	(0.001)	(0.084)	(0.004)	(0.06)
0.2	20	0.76	0.53	0.98	0.94	0.9	0.76	0.74	0.49	1	0.98	0.89	0.88	0.73	0.45	1	0.99	0.88	0.92
		(0.091)	(0.179)	(0.04)	(0.163)	(0.037)	(0.092)	(0.089)	(0.179)	(0.022)	(0.099)	(0.035)	(0.081)	(0.091)	(0.18)	(0.016)	(0.08)	(0.035)	(0.067)
	500	0.76	0.54	0.98	0.9	0.9	0.77	0.75	0.5	0.99	0.96	0.89	0.88	0.73	0.47	1	0.98	0.88	0.92
		(0.048)	(0.099)	(0.013)	(0.068)	(0.02)	(0.048)	(0.053)	(0.107)	(0.006)	(0.039)	(0.021)	(0.039)	(0.054)	(0.11)	(0.004)	(0.032)	(0.021)	(0.03)
0.3	20	0.85	0.74	0.96	0.92	0.9	0.85	0.87	0.75	0.98	0.96	0.91	0.94	0.87	0.75	0.99	0.97	0.9	0.97
		(0.083)	(0.158)	(0.064)	(0.134)	(0.056)	(0.079)	(0.079)	(0.156)	(0.039)	(0.09)	(0.054)	(0.053)	(0.08)	(0.157)	(0.035)	(0.081)	(0.055)	(0.04)
	500	0.85	0.74	0.96	0.9	0.9	0.85	0.87	0.75	0.98	0.95	0.9	0.94	0.87	0.74	0.99	0.97	0.9	0.97
		(0.033)	(0.07)	(0.02)	(0.047)	(0.024)	(0.035)	(0.035)	(0.073)	(0.012)	(0.03)	(0.025)	(0.019)	(0.036)	(0.076)	(0.009)	(0.024)	(0.026)	(0.013)
0.4	20	0.89	0.84	0.94	0.91	0.9	0.89	0.91	0.86	0.96	0.95	0.92	0.97	0.92	0.87	0.97	0.96	0.92	0.98
		(0.072)	(0.127)	(0.08)	(0.105)	(0.07)	(0.073)	(0.065)	(0.117)	(0.064)	(0.085)	(0.065)	(0.041)	(0.061)	(0.112)	(0.056)	(0.075)	(0.062)	(0.03)
	500	0.89	0.84	0.94	0.9	0.9	0.89	0.92	0.87	0.96	0.94	0.92	0.96	0.92	0.88	0.97	0.96	0.92	0.98
		(0.026)	(0.054)	(0.031)	(0.041)	(0.029)	(0.026)	(0.022)	(0.049)	(0.022)	(0.03)	(0.027)	(0.012)	(0.021)	(0.048)	(0.018)	(0.026)	(0.027)	(0.008)
0.5	20	0.9	0.9	0.9	0.9	0.91	0.9	0.93	0.93	0.93	0.93	0.93	0.97	0.94	0.94	0.94	0.95	0.95	0.98
		(0.071)	(0.106)	(0.1)	(0.087)	(0.089)	(0.07)	(0.06)	(0.087)	(0.084)	(0.074)	(0.076)	(0.038)	(0.052)	(0.08)	(0.074)	(0.065)	(0.069)	(0.027)
	500	0.9	0.9	0.9	0.9	0.9	0.9	0.93	0.93	0.93	0.93	0.93	0.97	0.94	0.94	0.94	0.94	0.94	0.98
		(0.022)	(0.039)	(0.042)	(0.035)	(0.033)	(0.023)	(0.017)	(0.031)	(0.034)	(0.029)	(0.027)	(0.011)	(0.015)	(0.028)	(0.03)	(0.027)	(0.025)	(0.007)

k_1^{train}	n_{test}	DLDA PA	DLDA PA_1	DLDA PA_2	DLDA PV_1	DLDA PV_2	DLDA AUC	DQDA PA	DQDA PA_1	DQDA PA_2	DQDA PV_1	DQDA PV_2	DQDA AUC	RF PA	RF PA_1	RF PA_2	RF PV_1	RF PV_2	RF AUC
0.1	20	0.64	0.28	1	0.97	0.93	0.94	0.56	0.12	1	NA	0.91	0.91	0.5	0	1	NA	0.9	0.88
		(0.082)	(0.163)	(0.019)	(0.145)	(0.016)	(0.058)	(0.056)	(0.112)	(0.009)	(NA)	(0.011)	(0.076)	(0.01)	(0.021)	(0)	(NA)	(0.002)	(0.083)
	500	0.64	0.28	1	0.92	0.93	0.94	0.56	0.11	1	0.94	0.91	0.91	0.5	0	1	NA	0.9	0.89
		(0.049)	(0.097)	(0.004)	(0.101)	(0.009)	(0.028)	(0.032)	(0.064)	(0.002)	(0.124)	(0.006)	(0.042)	(0.003)	(0.007)	(0)	(NA)	(0.001)	(0.038)
0.2	20	0.88	0.78	0.99	0.96	0.95	0.98	0.83	0.66	0.99	0.96	0.92	0.98	0.61	0.21	1	NA	0.84	0.96
		(0.072)	(0.143)	(0.034)	(0.11)	(0.032)	(0.024)	(0.088)	(0.173)	(0.032)	(0.116)	(0.037)	(0.031)	(0.072)	(0.144)	(0.008)	(NA)	(0.026)	(0.039)
	500	0.89	0.79	0.99	0.95	0.95	0.98	0.83	0.67	0.99	0.95	0.92	0.98	0.61	0.22	1	0.99	0.84	0.97
		(0.032)	(0.063)	(0.008)	(0.036)	(0.014)	(0.008)	(0.043)	(0.087)	(0.007)	(0.038)	(0.019)	(0.012)	(0.035)	(0.071)	(0.001)	(0.022)	(0.012)	(0.015)
0.3	20	0.94	0.91	0.98	0.96	0.96	0.99	0.92	0.86	0.98	0.96	0.94	0.99	0.8	0.61	1	0.99	0.86	0.98
		(0.052)	(0.095)	(0.045)	(0.088)	(0.037)	(0.019)	(0.061)	(0.115)	(0.043)	(0.088)	(0.044)	(0.023)	(0.084)	(0.168)	(0.014)	(0.04)	(0.053)	(0.027)
	500	0.94	0.91	0.98	0.95	0.96	0.99	0.92	0.86	0.98	0.95	0.94	0.99	0.8	0.61	1	0.99	0.86	0.98
		(0.016)	(0.031)	(0.011)	(0.025)	(0.012)	(0.004)	(0.022)	(0.045)	(0.011)	(0.025)	(0.017)	(0.005)	(0.033)	(0.067)	(0.004)	(0.015)	(0.021)	(0.007)
0.4	20	0.96	0.94	0.97	0.96	0.96	0.99	0.95	0.93	0.97	0.96	0.96	0.99	0.91	0.84	0.99	0.98	0.91	0.99
		(0.047)	(0.075)	(0.052)	(0.069)	(0.046)	(0.015)	(0.051)	(0.085)	(0.054)	(0.071)	(0.051)	(0.017)	(0.063)	(0.122)	(0.038)	(0.056)	(0.065)	(0.022)
	500	0.96	0.94	0.97	0.96	0.96	0.99	0.95	0.93	0.97	0.96	0.95	0.99	0.91	0.84	0.99	0.98	0.9	0.99
		(0.011)	(0.022)	(0.013)	(0.019)	(0.014)	(0.003)	(0.014)	(0.029)	(0.014)	(0.02)	(0.017)	(0.004)	(0.021)	(0.043)	(0.01)	(0.016)	(0.024)	(0.005)
0.5	20	0.96	0.96	0.96	0.96	0.96	0.99	0.95	0.95	0.95	0.96	0.96	0.99	0.95	0.95	0.95	0.95	0.95	0.99
		(0.044)	(0.063)	(0.063)	(0.056)	(0.056)	(0.016)	(0.046)	(0.067)	(0.068)	(0.059)	(0.059)	(0.018)	(0.048)	(0.068)	(0.071)	(0.063)	(0.061)	(0.019)
	500	0.96	0.96	0.96	0.96	0.96	0.99	0.95	0.95	0.95	0.96	0.96	0.99	0.95	0.95	0.95	0.95	0.95	0.99
		(0.01)	(0.016)	(0.017)	(0.016)	(0.015)	(0.003)	(0.011)	(0.02)	(0.019)	(0.018)	(0.018)	(0.004)	(0.014)	(0.023)	(0.021)	(0.02)	(0.021)	(0.005)

k_1^{train}	n_{test}	SVM PA	SVM PA_1	SVM PA_2	SVM PV_1	SVM PV_2	SVM AUC	PAM PA	PAM PA_1	PAM PA_2	PAM PV_1	PAM PV_2	PAM AUC	PLR PA	PLR PA_1	PLR PA_2	PLR PV_1	PLR PV_2	PLR AUC
0.1	20	0.52	0.03	1	NA	0.9	0.94	0.52	0.04	1	NA	0.9	0.96	0.53	0.07	1	NA	0.91	0.94
		(0.029)	(0.057)	(0)	(NA)	(0.005)	(0.059)	(0.031)	(0.061)	(0)	(NA)	(0.006)	(0.049)	(0.041)	(0.083)	(0.003)	(NA)	(0.008)	(0.06)
	500	0.52	0.03	1	0.99	0.9	0.94	0.52	0.04	1	1	0.9	0.96	0.53	0.07	1	0.99	0.91	0.94
		(0.011)	(0.023)	(0)	(0.069)	(0.002)	(0.03)	(0.016)	(0.033)	(0)	(0.036)	(0.003)	(0.025)	(0.021)	(0.042)	(0.001)	(0.07)	(0.004)	(0.031)
0.2	20	0.75	0.51	1	0.99	0.89	0.98	0.74	0.48	1	1	0.89	0.99	0.8	0.6	1	0.99	0.91	0.98
		(0.089)	(0.177)	(0.014)	(0.064)	(0.035)	(0.025)	(0.092)	(0.185)	(0.006)	(0.033)	(0.036)	(0.018)	(0.088)	(0.174)	(0.018)	(0.08)	(0.036)	(0.025)
	500	0.76	0.52	1	0.98	0.89	0.98	0.74	0.49	1	1	0.89	0.99	0.8	0.6	1	0.98	0.91	0.98
		(0.045)	(0.09)	(0.003)	(0.024)	(0.018)	(0.009)	(0.049)	(0.099)	(0.001)	(0.011)	(0.019)	(0.006)	(0.044)	(0.089)	(0.004)	(0.023)	(0.018)	(0.009)
0.3	20	0.9	0.81	0.99	0.98	0.93	0.99	0.89	0.79	1	1	0.92	0.99	0.91	0.83	0.99	0.98	0.93	0.99
		(0.067)	(0.132)	(0.028)	(0.063)	(0.048)	(0.02)	(0.069)	(0.138)	(0.015)	(0.032)	(0.049)	(0.013)	(0.064)	(0.125)	(0.034)	(0.07)	(0.046)	(0.019)
	500	0.9	0.81	0.99	0.98	0.93	0.99	0.89	0.79	1	0.99	0.92	1	0.91	0.84	0.99	0.97	0.93	0.99
		(0.026)	(0.053)	(0.007)	(0.017)	(0.019)	(0.005)	(0.029)	(0.058)	(0.003)	(0.009)	(0.021)	(0.003)	(0.024)	(0.048)	(0.007)	(0.019)	(0.018)	(0.005)
0.4	20	0.95	0.92	0.98	0.97	0.95	0.99	0.95	0.91	0.99	0.99	0.95	1	0.95	0.92	0.98	0.97	0.95	0.99
		(0.051)	(0.091)	(0.044)	(0.061)	(0.053)	(0.016)	(0.051)	(0.099)	(0.027)	(0.038)	(0.056)	(0.011)	(0.052)	(0.09)	(0.049)	(0.066)	(0.053)	(0.015)
	500	0.95	0.92	0.98	0.97	0.95	0.99	0.95	0.91	0.99	0.99	0.94	1	0.95	0.92	0.98	0.97	0.95	0.99
		(0.015)	(0.03)	(0.011)	(0.017)	(0.018)	(0.004)	(0.019)	(0.039)	(0.007)	(0.011)	(0.022)	(0.002)	(0.016)	(0.031)	(0.012)	(0.018)	(0.018)	(0.004)
0.5	20	0.96	0.96	0.96	0.96	0.96	0.99	0.96	0.96	0.96	0.97	0.96	0.99	0.96	0.96	0.96	0.96	0.96	0.99
		(0.045)	(0.062)	(0.064)	(0.057)	(0.055)	(0.017)	(0.045)	(0.063)	(0.063)	(0.056)	(0.056)	(0.016)	(0.046)	(0.065)	(0.066)	(0.058)	(0.058)	(0.016)
	500	0.96	0.96	0.96	0.96	0.96	0.99	0.96	0.96	0.96	0.96	0.96	0.99	0.96	0.96	0.96	0.96	0.96	0.99
		(0.011)	(0.018)	(0.018)	(0.017)	(0.017)	(0.003)	(0.019)	(0.023)	(0.022)	(0.021)	(0.022)	(0.007)	(0.012)	(0.019)	(0.02)	(0.019)	(0.018)	(0.003)

Table 1: Predictive accuracy (PA), class 1 PA (PA_1), class 2 PA (PA_2), positive and negative predictive value (PV_1, PV_2) for different proportions of class 1 samples in the training set (k_1^{train}, $n_{train} = 80$). Test set was balanced and contained 20 or 500 samples (n_{test}). 1000 variables were simulated from $N(0,1)$ for all class 1 samples and for class 2 samples 20 variables were simulated from $N(1,1)$ distribution and others from $N(0,1)$. 40 variables with largest t-statistic were selected and used for classification.
