# Supplementary material for: Class prediction for high-dimensional class-imbalanced data
Source: BMC Bioinformatics. 2010 Oct 20;11:523. doi: 10.1186/1471-2105-11-523 (PMC3098087; doi:10.1186/1471-2105-11-523)
Supplement: Additional file 4 — Effect of performing variable selection and increasing the number of variables for the nine classifiers. The additional file reports the same results described in the left panels of Figure 3 for 1-NN, DLDA and PLR, but for all the classifiers. [file 1471-2105-11-523-S4.PDF]

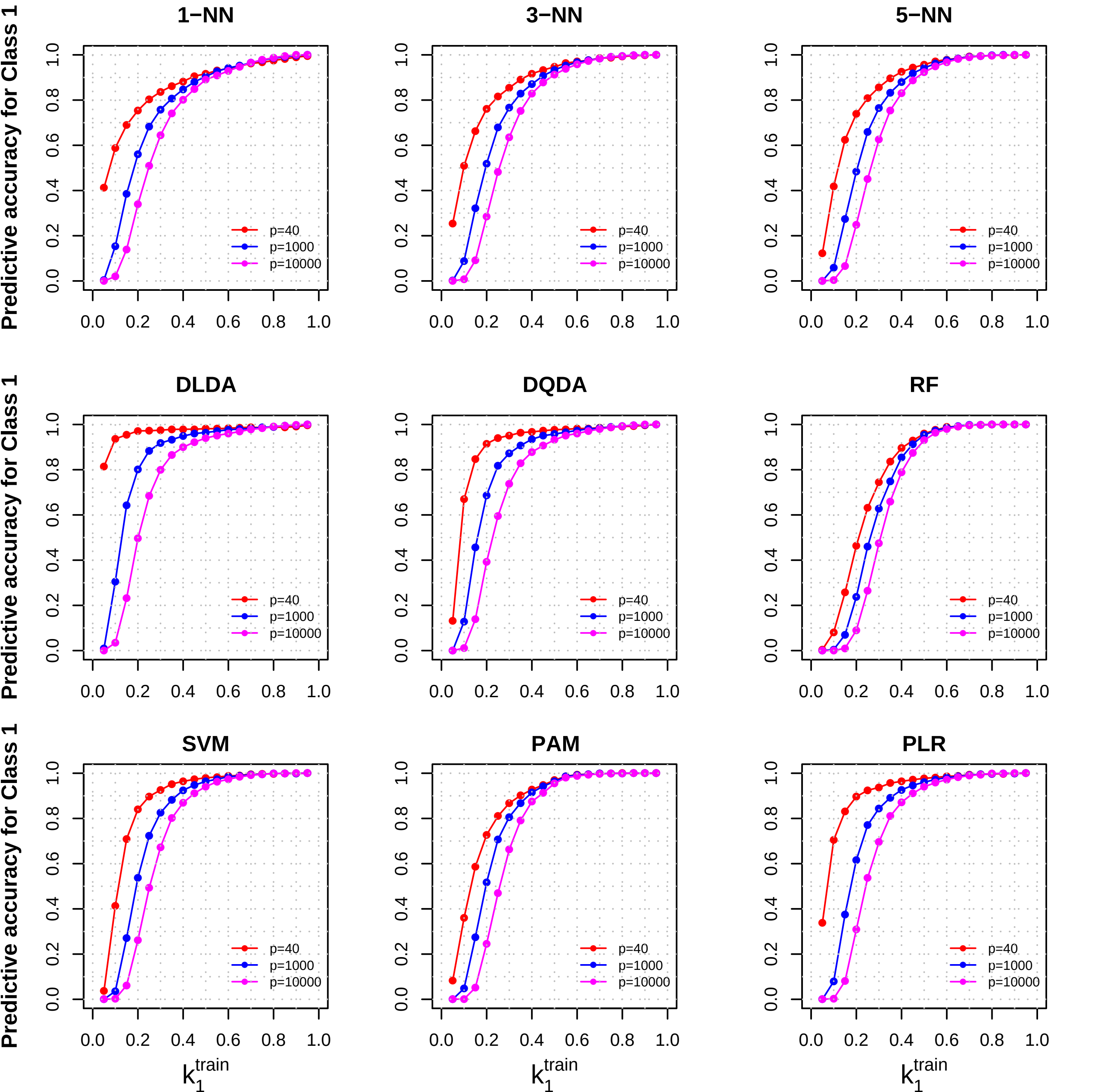
1−NN
3−NN
5−NN
DLDA
DQDA
RF
SVM
PAM
PLR
Predictive accuracy for Class 1
p=40
p=1000
p=10000
k_1^{train}
