# Supplementary material for: Class prediction for high-dimensional class-imbalanced data
Source: BMC Bioinformatics. 2010 Oct 20;11:523. doi: 10.1186/1471-2105-11-523 (PMC3098087; doi:10.1186/1471-2105-11-523)
Supplement: Additional file 5 — Effect of performing variable selection and increasing the number of variables when all the variables are different between the two classes, for the nine classifiers. The additional file reports the same results described in the right panels of Figure 3 for 1-NN, DLDA and PLR, but for all the classifiers. [file 1471-2105-11-523-S5.PDF]

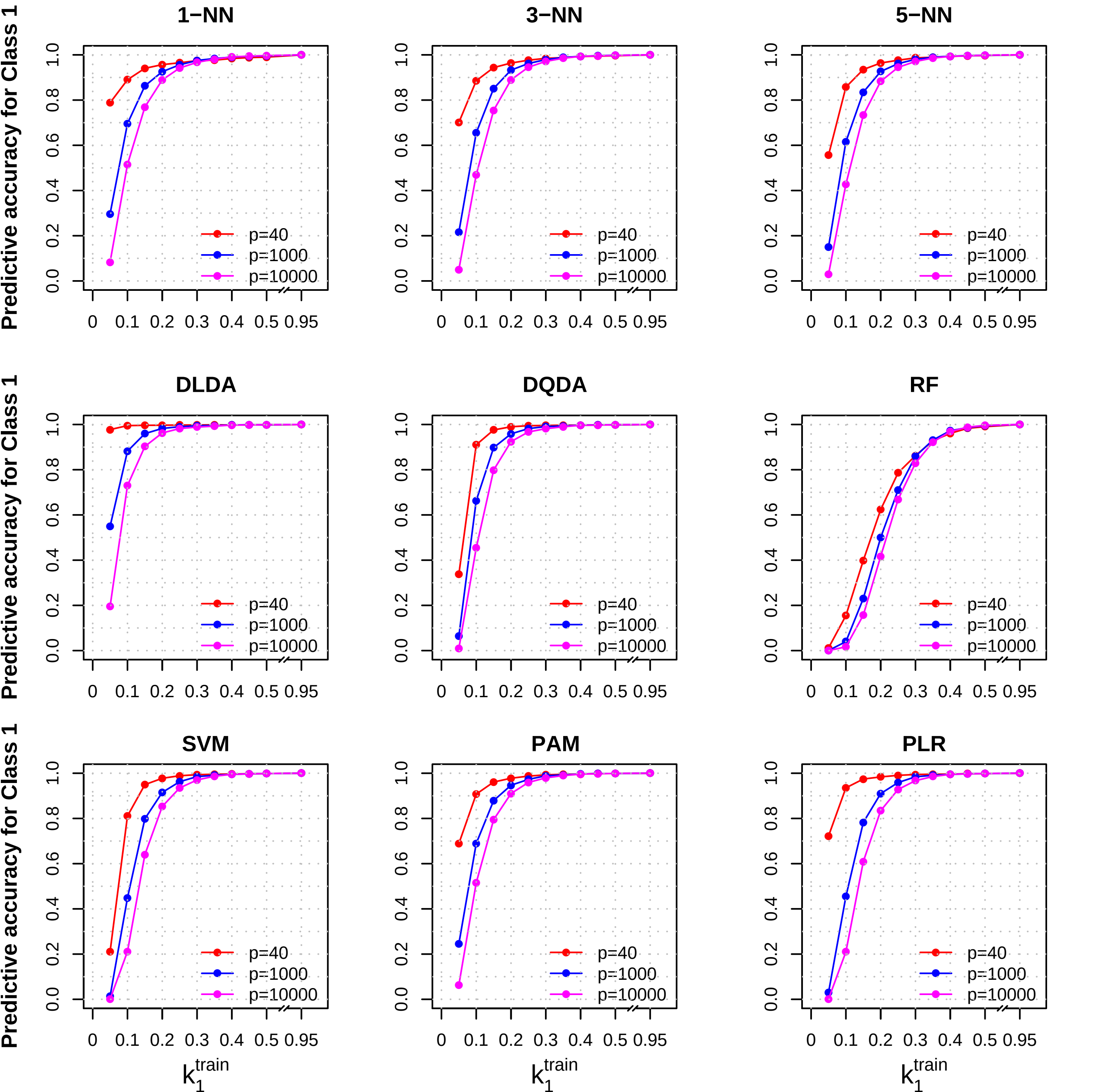
1−NN
3−NN
5−NN
DLDA
DQDA
RF
SVM
PAM
PLR
Predictive accuracy for Class 1
p=40
p=1000
p=10000
0.0 0.2 0.4 0.6 0.8 1.0
0 0.1 0.2 0.3 0.4 0.5 0.95
k_1^{train}
