# Supplementary figures and images for: Class prediction for high-dimensional class-imbalanced data
Source: BMC Bioinformatics. 2010 Oct 20;11:523. doi: 10.1186/1471-2105-11-523 (PMC3098087; doi:10.1186/1471-2105-11-523)

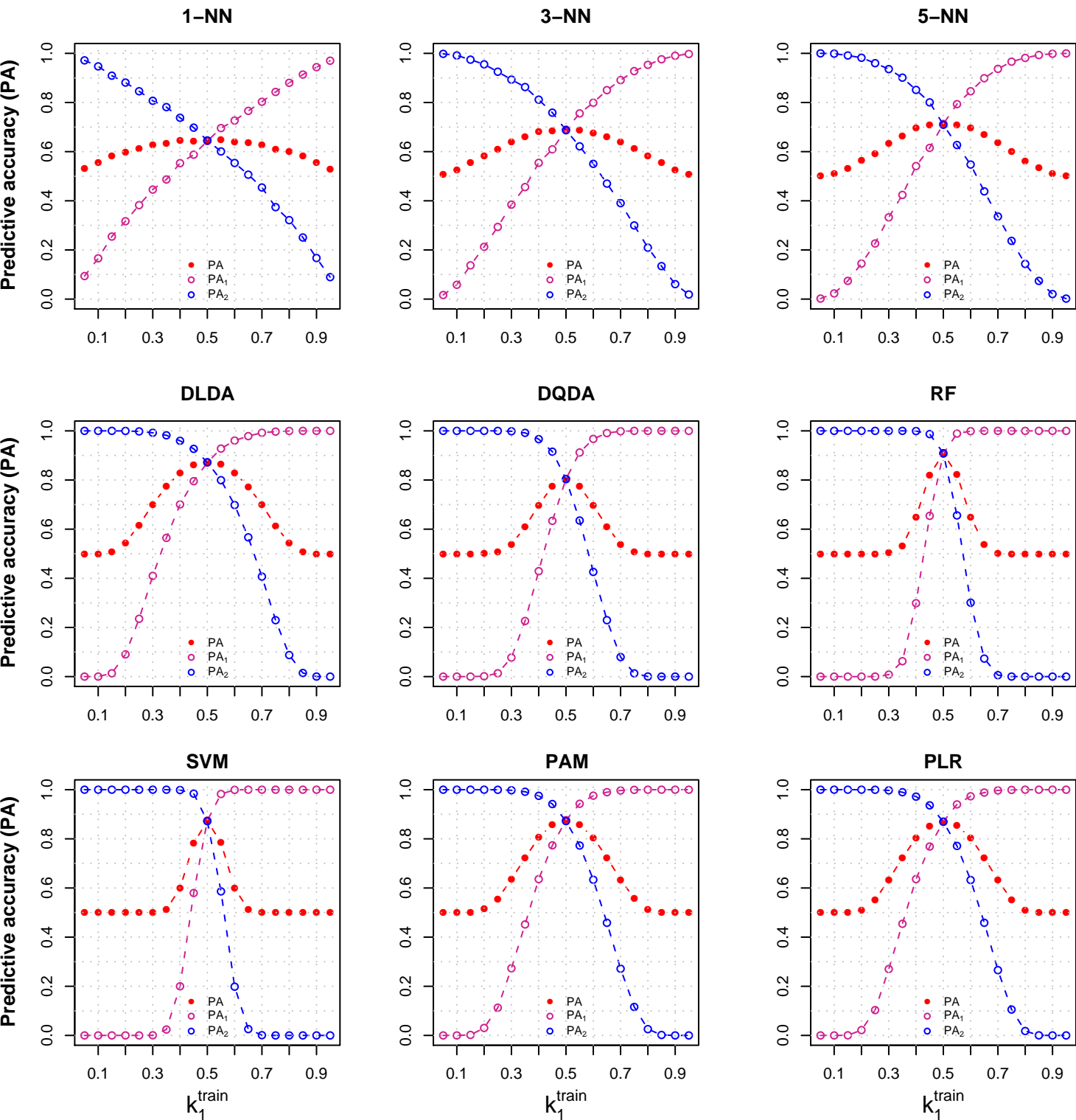

Supplement: Additional file 6 — Effect of not performing variable selection. The additional file reports the predictive accuracy results obtained for the nine classifiers, when the number of variables is large (p = 1000) and variable selection is not performed. The simulation setting is the same as for Figure 5, first column. [file 1471-2105-11-523-S6.PDF]

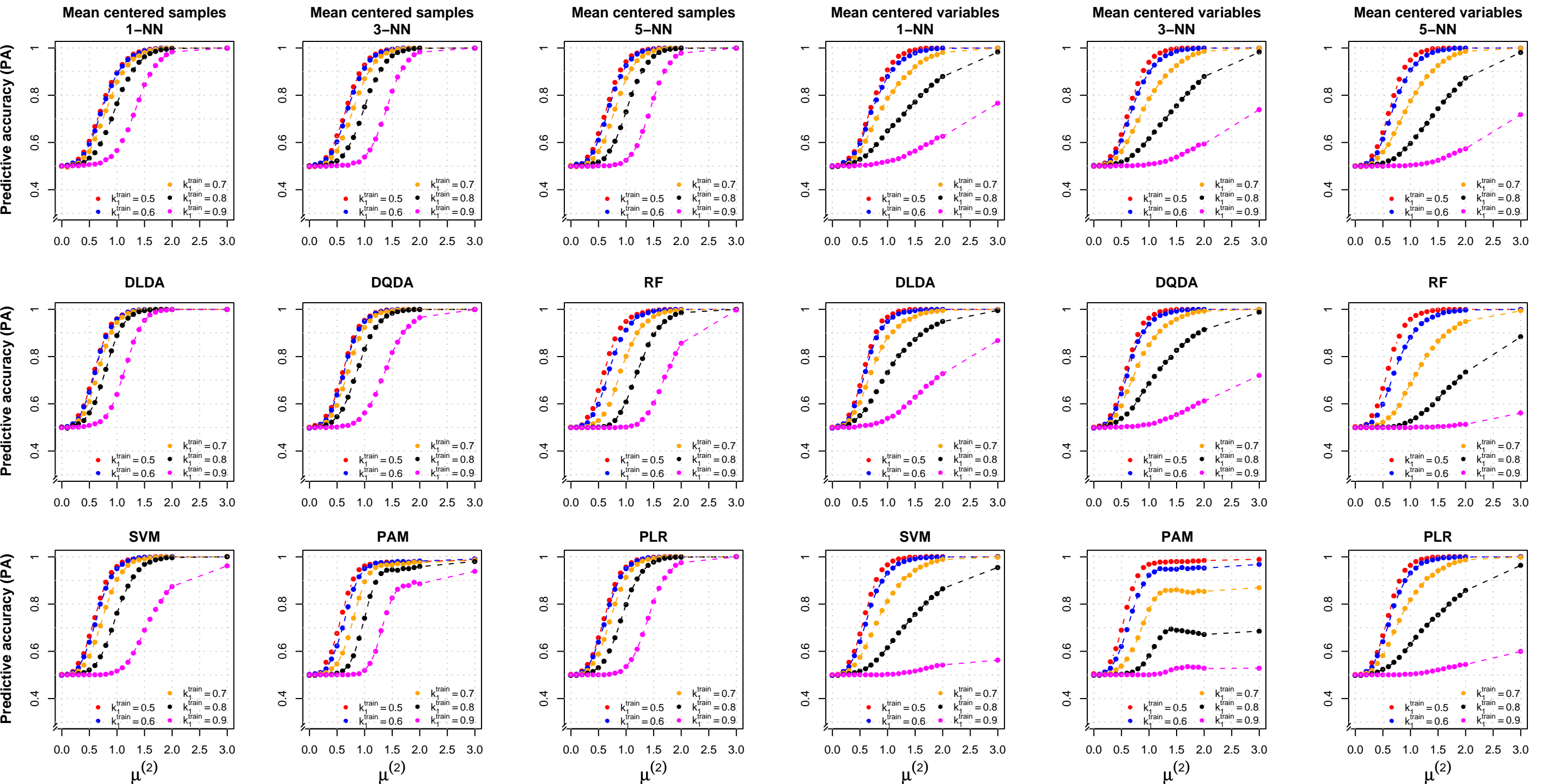

Supplement: Additional file 7 — Effect of varying the magnitude of the difference between classes. The additional file shows, for the nine classifiers, the same results presented in Figure 4 for 1-NN, DLDA and PLR. [file 1471-2105-11-523-S7.PDF]

## No correction

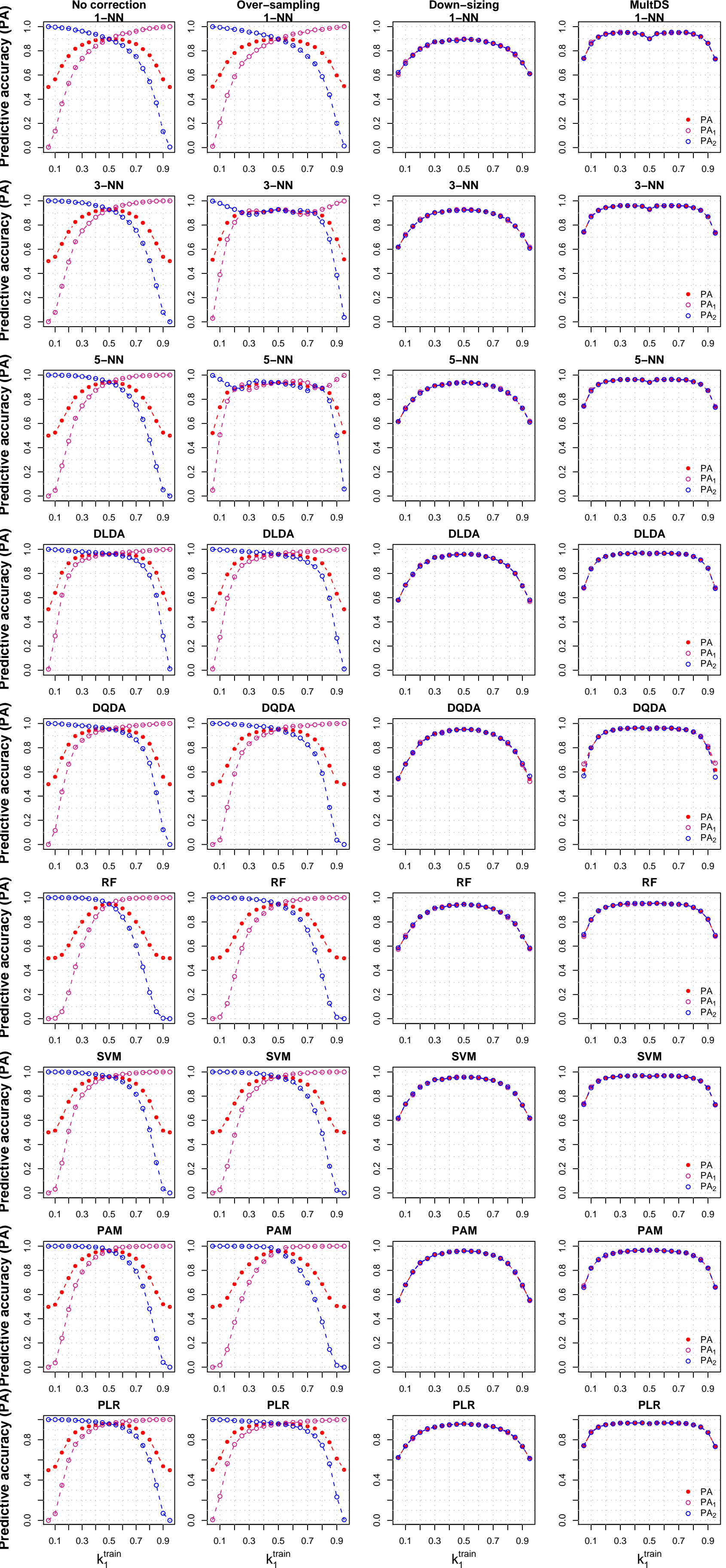

Supplement: Additional file 8 — Solutions to the class imbalance problem. The additional file shows, for the nine classifiers, the same results presented in Figure 5 for 3-NN, DLDA and PLR. [file 1471-2105-11-523-S8.PDF]

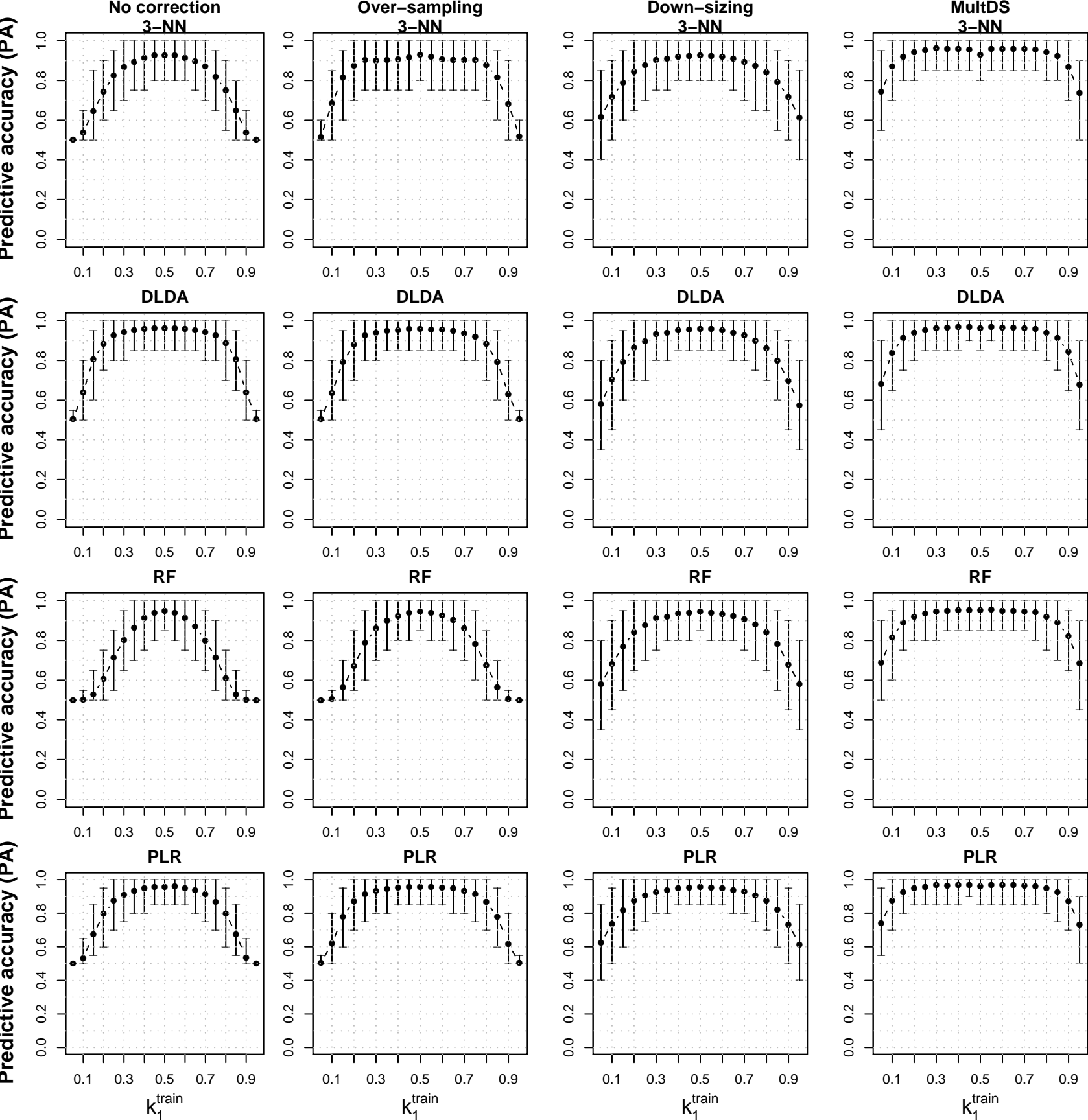

Supplement: Additional file 10 — Variability of the overall predictive accuracy. The figure shows, for four classifiers, the overall predictive accuracy and its 95% prediction intervals (obtained with no correction, over-sampling, down-sizing and multiple down-sizing). The simulation setting is the same as described for Figure 5, but the test set contained 500 samples. [file 1471-2105-11-523-S10.PDF]

RF

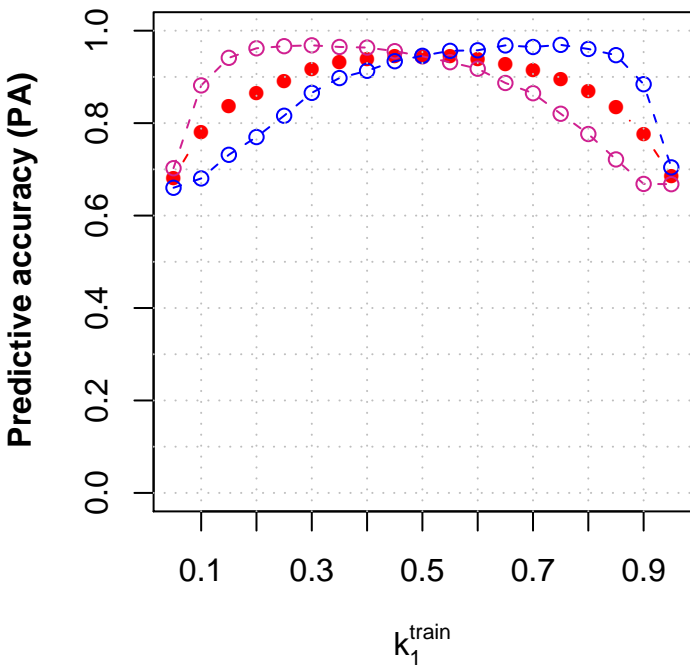

PLR

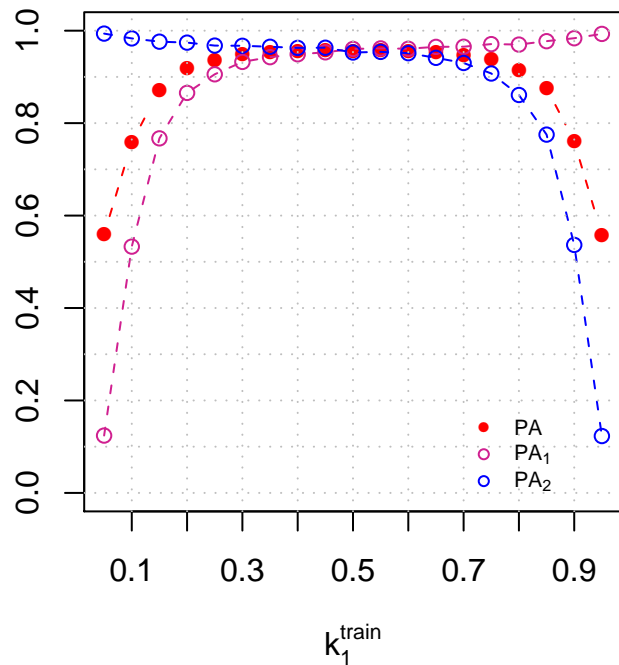

Supplement: Additional file 11 — Threshold based on class imbalance for classification with PLR and RF. The figure shows, for RF and PLR, the overall (PA) and class specific predictive accuracy (PA1 and PA2) obtained in the same simulation setting of Figure 5: here the classification rule was based on the threshold equal to the class imbalance of the training set (RF-THR and PLR-THR). [file 1471-2105-11-523-S11.PDF]

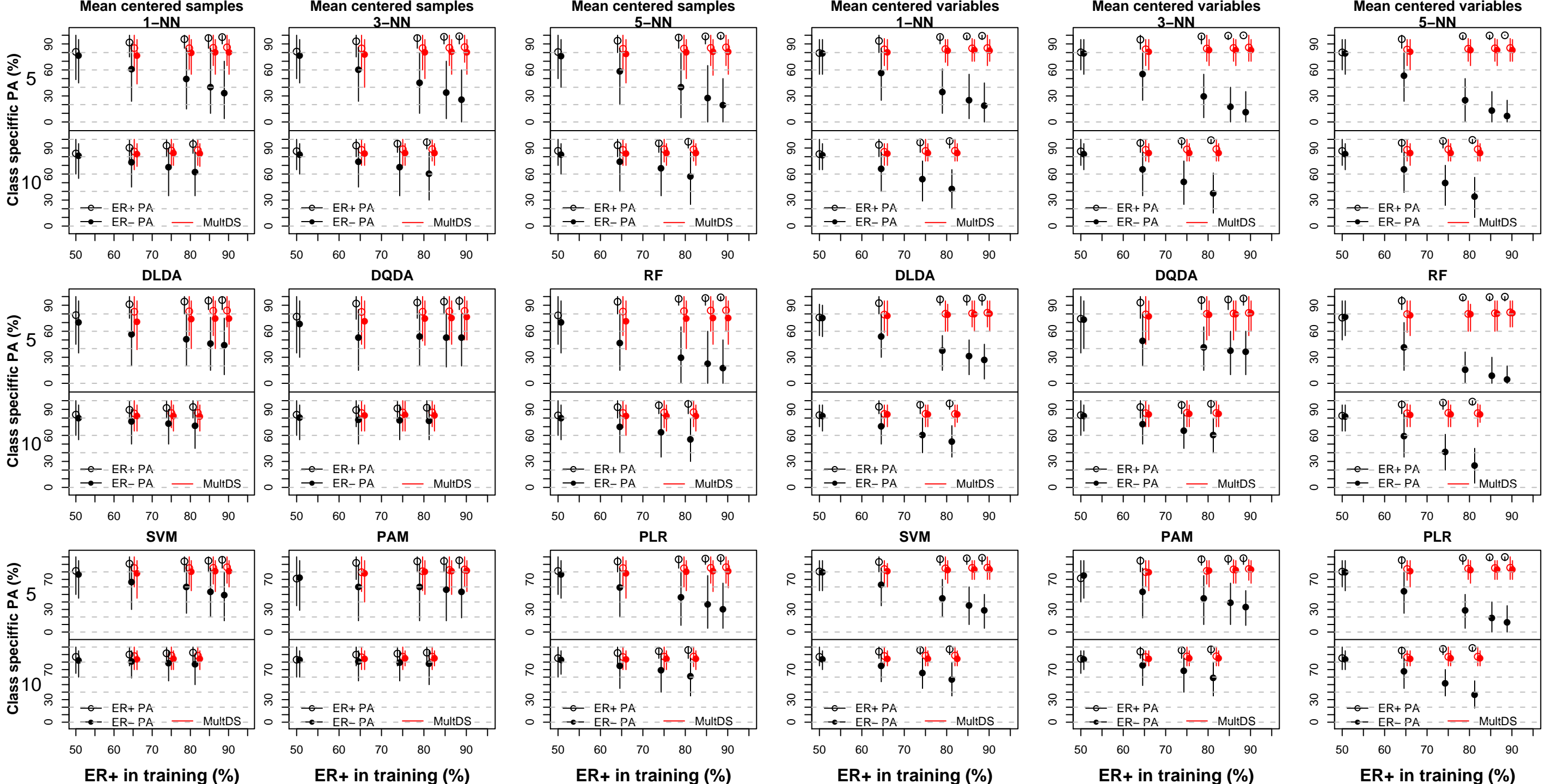

Supplement: Additional file 12 — Overall predictive accuracy and the 95% prediction intervals for the prediction of ER status. The figure shows for all the classifiers the same results as those presented in Figure 6. [file 1471-2105-11-523-S12.PDF]
