# Supplementary material for: Class prediction for high-dimensional class-imbalanced data
Source: BMC Bioinformatics. 2010 Oct 20;11:523. doi: 10.1186/1471-2105-11-523 (PMC3098087; doi:10.1186/1471-2105-11-523)
Supplement: Additional file 9 — Over-sampling and variable centering. The additional file shows the behavior of the classifiers using over-sampling and mean-centering the variables. An explanation for the improved behavior of DLDA is given. [file 1471-2105-11-523-S9.PDF]

Figure 1 shows overall (PA) and class specific predictive accuracy of the calssifiers (PA_1 and PA_2), varying the proportion Class 1 samples in training set (k_1^{train}). One thousand variables were generated from standard normal distribution ($N(0, 1)$) for the samples from Class 1, while for Class 2 20 variables were generated from $N(1, 1)$, and the remaining 980 variables from $N(0, 1)$. The 40 variables with the highest absolute value of univariate t-statistic were selected and used in classification. We obtained a balanced training set by replicating the samples from the minority class (over-sampling); the variables were mean-centered after over-sampling. The test set was either balanced ($k_1^{test} = 0.5$) or equally imbalanced as the training set ($k_1^{test} = k_1^{train}$). When the test set was balanced ($k_1^{test} = 0.5$) the performance of 1-NN, DLDA and PLR was improved: the discrepancy between the class-specific predictive accuracies were smaller, and completely disappeared for DLDA. Some improvements were seen for RF, SVM and PAM when $k_1^{test} = k_1^{train}$, however only if the class imbalance was not too extreme.

To some extent the surprising behavior of DLDA can be explained by looking at Figure 2. We used the following simulation settings when constructing the figure. One variable was generated from $N(0, 1)$ for 4 Class 1 samples and one from $N(1, 1)$ for 76 Class 2 samples ($k_1^{train} = 0.05$, $n_{train} = 80$), while the test set was balanced (10 samples per class) and the variable was simulated from the same distribution as in the training set. Over-sampling was performed (with 19 replications of the minority class). We show the distribution of the sample means in Class 1 and Class 2 in the first part of the plot, and the distribution of the class specific sample means after over-sampling in the second part of the plot. The third part of the plot shows the distribution of the sample means in the test set. The lower panel of the plot shows the distribution of the sample means obtained mean-centering the variables. When the variables are mean centered, the sample means are shifted towards the mean of the larger class (note that since we have such a large imbalance, the overall mean of the variable is closer to the mean of the larger class), while in the test set the sample means are shifted towards the mid point of two class means. Furthermore, the variability of the sample means in the larger class is heavily reduced. It is obvious that the samples from the larger class in the test set will be classified in the larger class in most cases. It is also straightforward to see that due to mean-centering, the smaller class becomes more similar to the larger class in the training set, therefore the new samples from the smaller class will be classified in the larger class. When over-sampling is used it can be seen that the training set becomes very similar to the test set, therefore more samples are classified correctly. Since the variables were generated independently and the classification rule for DLDA sums up the Mahalanobis distances for each variable, this effect is even more obvious in the high-dimensional setting.

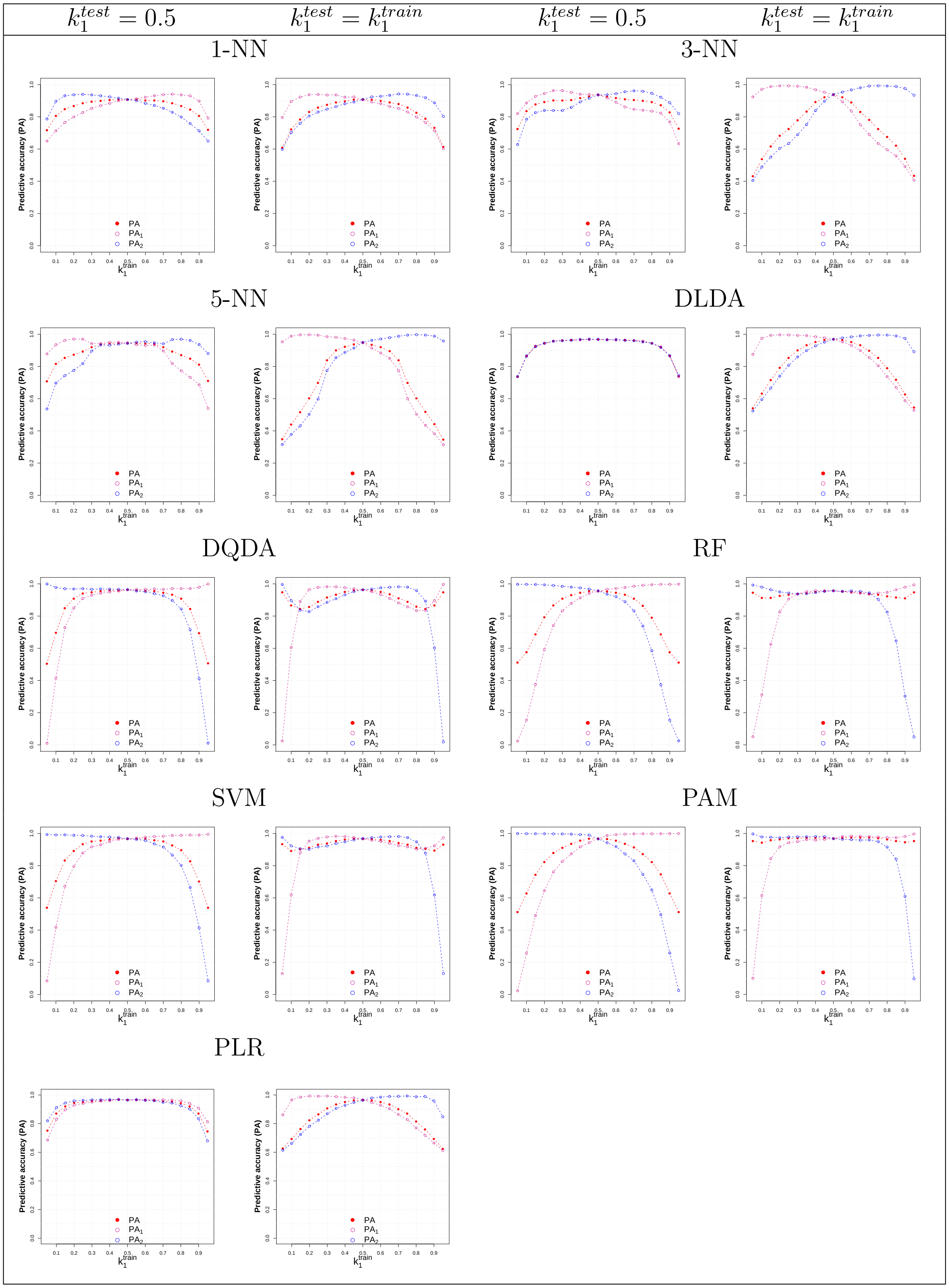

Figure 1: Predictive accuracy (PA), class 1 PA (PA_1) and class 2 PA (PA_2) for different classification methods. Smaller class was over-sampled and variables were mean centered.

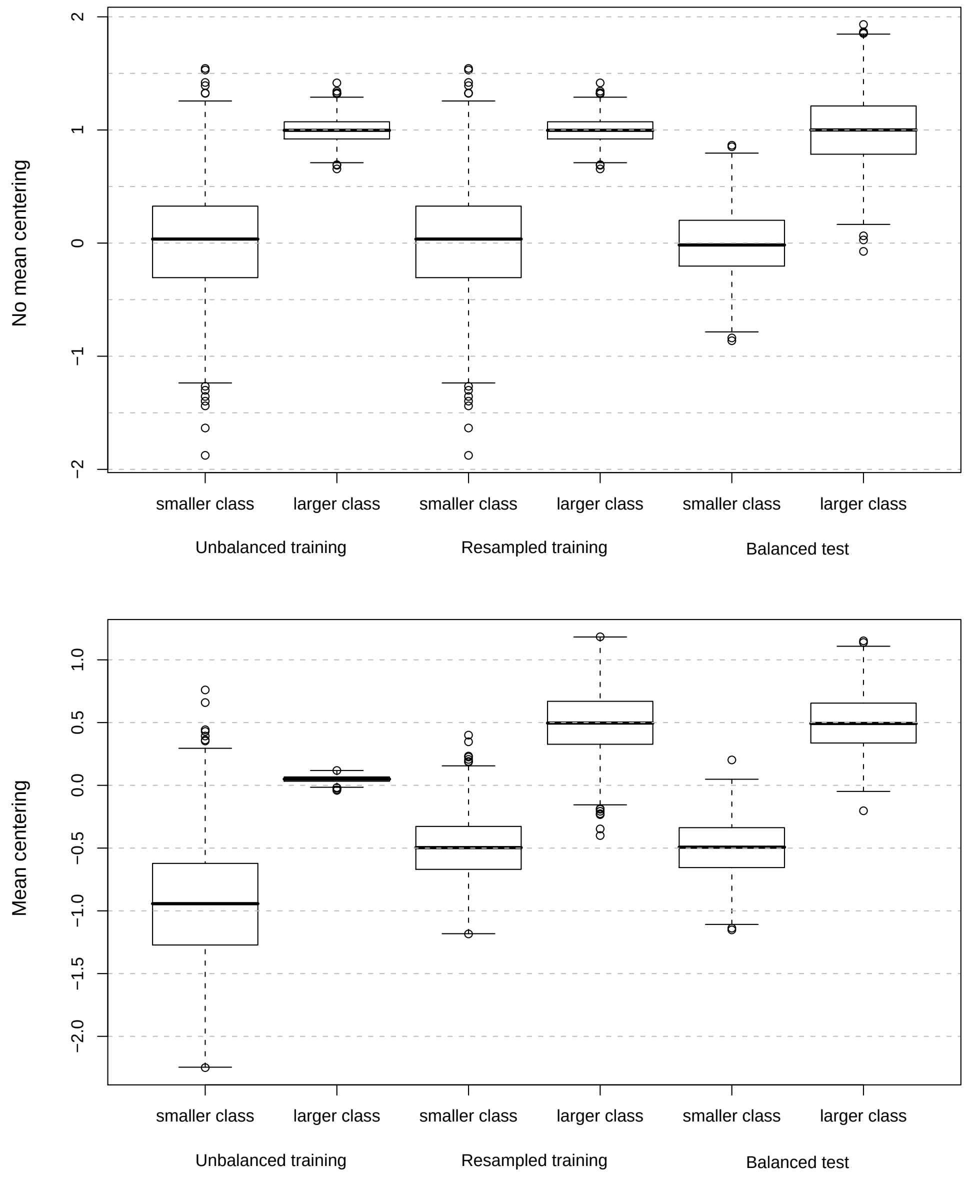

Figure 2: Distribution of the sample mean
