# Supplementary material for: Class prediction for high-dimensional class-imbalanced data
Source: BMC Bioinformatics. 2010 Oct 20;11:523. doi: 10.1186/1471-2105-11-523 (PMC3098087; doi:10.1186/1471-2105-11-523)
Supplement: Additional file 13 — Behavior of the nine classifiers for the prediction of ER status - no correction. The table shows, for all the classifiers, the predictive accuracies presented in Figure 6 (no correction for class imbalance), together with the predictive values and AUC. [file 1471-2105-11-523-S13.PDF]

ER+	ER-	k^{train}_{ER+}	1-NN						3-NN						5-NN					
			PA	PA_{ER+}	PA_{ER-}	PV_{ER+}	PV_{ER-}	AUC	PA	PA_{ER+}	PA_{ER-}	PV_{ER+}	PV_{ER-}	AUC	PA	PA_{ER+}	PA_{ER-}	PV_{ER+}	PV_{ER-}	AUC
5	5	0.5	0.78	0.81	0.75	0.78	0.81	0.78	0.79	0.82	0.75	0.78	0.82	0.82	0.78	0.81	0.76	0.78	0.81	0.84
			(0.09)	(0.14)	(0.15)	(0.1)	(0.11)	(0.09)	(0.09)	(0.14)	(0.15)	(0.1)	(0.11)	(0.09)	(0.09)	(0.14)	(0.16)	(0.1)	(0.11)	(0.09)
10		0.67	0.76	0.92	0.61	0.83	0.8	0.77	0.77	0.93	0.6	0.83	0.83	0.82	0.76	0.94	0.58	0.82	0.84	0.84
			(0.09)	(0.07)	(0.18)	(0.07)	(0.14)	(0.09)	(0.09)	(0.07)	(0.19)	(0.07)	(0.14)	(0.09)	(0.1)	(0.06)	(0.2)	(0.07)	(0.14)	(0.08)
20		0.8	0.72	0.95	0.49	0.88	0.78	0.73	0.71	0.97	0.45	0.88	0.83	0.8	0.69	0.97	0.4	0.87	0.84	0.82
			(0.09)	(0.05)	(0.19)	(0.04)	(0.21)	(0.09)	(0.09)	(0.04)	(0.19)	(0.04)	(0.21)	(0.09)	(0.09)	(0.04)	(0.19)	(0.04)	(0.22)	(0.08)
30		0.86	0.68	0.97	0.4	0.91	0.78	0.69	0.66	0.98	0.34	0.9	0.85	0.76	0.63	0.99	0.28	0.89	0.88	0.79
			(0.09)	(0.04)	(0.19)	(0.03)	(0.26)	(0.09)	(0.09)	(0.03)	(0.18)	(0.02)	(0.25)	(0.09)	(0.08)	(0.03)	(0.17)	(0.02)	(0.24)	(0.09)
45		0.9	0.66	0.98	0.33	0.93	0.79	0.66	0.62	0.99	0.26	0.92	0.88	0.73	0.59	0.99	0.2	0.92	NaN	0.76
			(0.09)	(0.03)	(0.18)	(0.02)	(0.3)	(0.08)	(0.08)	(0.03)	(0.16)	(0.02)	(0.25)	(0.09)	(0.07)	(0.02)	(0.15)	(0.01)	(NA)	(0.09)
10	10	0.5	0.82	0.83	0.8	0.82	0.84	0.82	0.84	0.86	0.81	0.83	0.86	0.88	0.84	0.87	0.82	0.83	0.87	0.89
			(0.07)	(0.11)	(0.1)	(0.07)	(0.09)	(0.07)	(0.06)	(0.09)	(0.09)	(0.07)	(0.08)	(0.05)	(0.06)	(0.09)	(0.09)	(0.06)	(0.08)	(0.05)
20		0.67	0.82	0.9	0.74	0.88	0.81	0.82	0.84	0.93	0.74	0.88	0.85	0.87	0.84	0.93	0.74	0.88	0.86	0.89
			(0.07)	(0.07)	(0.13)	(0.05)	(0.12)	(0.07)	(0.06)	(0.06)	(0.13)	(0.05)	(0.11)	(0.05)	(0.07)	(0.06)	(0.13)	(0.05)	(0.11)	(0.05)
30		0.75	0.8	0.93	0.68	0.9	0.79	0.8	0.82	0.95	0.68	0.9	0.85	0.87	0.81	0.96	0.67	0.9	0.86	0.88
			(0.06)	(0.06)	(0.14)	(0.04)	(0.14)	(0.07)	(0.07)	(0.05)	(0.15)	(0.04)	(0.14)	(0.05)	(0.07)	(0.05)	(0.15)	(0.04)	(0.13)	(0.04)
45		0.82	0.78	0.95	0.62	0.92	0.76	0.78	0.79	0.97	0.6	0.92	0.84	0.86	0.77	0.97	0.57	0.91	0.86	0.88
			(0.07)	(0.05)	(0.14)	(0.03)	(0.18)	(0.07)	(0.07)	(0.04)	(0.15)	(0.03)	(0.17)	(0.06)	(0.08)	(0.04)	(0.15)	(0.03)	(0.17)	(0.05)

ER+	ER-	k^{train}_{ER+}	DLDA						DQDA						RF					
			PA	PA_{ER+}	PA_{ER-}	PV_{ER+}	PV_{ER-}	AUC	PA	PA_{ER+}	PA_{ER-}	PV_{ER+}	PV_{ER-}	AUC	PA	PA_{ER+}	PA_{ER-}	PV_{ER+}	PV_{ER-}	AUC
5	5	0.5	0.74	0.79	0.69	0.73	0.78	0.81	0.72	0.77	0.68	0.72	0.77	0.79	0.75	0.79	0.7	0.74	0.78	0.82
			(0.09)	(0.14)	(0.16)	(0.1)	(0.12)	(0.1)	(0.1)	(0.18)	(0.18)	(0.11)	(0.13)	(0.1)	(0.09)	(0.14)	(0.15)	(0.1)	(0.11)	(0.09)
10		0.67	0.74	0.91	0.56	0.81	0.78	0.84	0.72	0.92	0.52	0.8	0.8	0.83	0.7	0.94	0.46	0.78	0.81	0.85
			(0.09)	(0.07)	(0.17)	(0.06)	(0.15)	(0.08)	(0.09)	(0.08)	(0.18)	(0.06)	(0.17)	(0.08)	(0.09)	(0.06)	(0.18)	(0.06)	(0.16)	(0.08)
20		0.8	0.72	0.94	0.51	0.89	0.74	0.86	0.73	0.93	0.54	0.89	0.72	0.86	0.64	0.97	0.3	0.85	0.81	0.87
			(0.08)	(0.06)	(0.16)	(0.03)	(0.21)	(0.06)	(0.08)	(0.07)	(0.17)	(0.03)	(0.21)	(0.08)	(0.08)	(0.04)	(0.17)	(0.03)	(0.26)	(0.06)
30		0.86	0.71	0.95	0.46	0.91	0.7	0.87	0.73	0.94	0.53	0.92	0.68	0.86	0.6	0.98	0.23	0.88	0.81	0.87
			(0.08)	(0.05)	(0.15)	(0.02)	(0.25)	(0.06)	(0.08)	(0.06)	(0.16)	(0.02)	(0.24)	(0.07)	(0.08)	(0.03)	(0.16)	(0.02)	(0.3)	(0.06)
45		0.9	0.7	0.96	0.44	0.94	0.69	0.88	0.74	0.95	0.53	0.95	0.66	0.88	0.58	0.99	0.18	0.92	NaN	0.87
			(0.07)	(0.05)	(0.16)	(0.02)	(0.3)	(0.05)	(0.08)	(0.05)	(0.15)	(0.02)	(0.29)	(0.06)	(0.07)	(0.02)	(0.14)	(0.01)	(NA)	(0.05)
10	10	0.5	0.82	0.84	0.79	0.81	0.84	0.88	0.82	0.84	0.79	0.81	0.85	0.88	0.81	0.84	0.79	0.8	0.84	0.88
			(0.06)	(0.1)	(0.1)	(0.07)	(0.08)	(0.05)	(0.06)	(0.12)	(0.11)	(0.08)	(0.09)	(0.06)	(0.07)	(0.11)	(0.1)	(0.07)	(0.09)	(0.05)
20		0.67	0.83	0.89	0.76	0.88	0.8	0.89	0.84	0.89	0.78	0.89	0.8	0.9	0.81	0.93	0.7	0.86	0.84	0.9
			(0.06)	(0.08)	(0.11)	(0.04)	(0.12)	(0.05)	(0.06)	(0.08)	(0.1)	(0.04)	(0.11)	(0.05)	(0.07)	(0.06)	(0.14)	(0.05)	(0.11)	(0.05)
30		0.75	0.83	0.92	0.74	0.91	0.77	0.9	0.84	0.91	0.77	0.92	0.77	0.91	0.79	0.95	0.64	0.89	0.83	0.91
			(0.06)	(0.06)	(0.11)	(0.03)	(0.14)	(0.04)	(0.05)	(0.07)	(0.1)	(0.03)	(0.14)	(0.04)	(0.07)	(0.05)	(0.14)	(0.04)	(0.15)	(0.04)
45		0.82	0.82	0.93	0.71	0.94	0.72	0.91	0.84	0.92	0.77	0.95	0.72	0.92	0.76	0.96	0.55	0.91	0.81	0.91
			(0.06)	(0.06)	(0.12)	(0.02)	(0.17)	(0.04)	(0.05)	(0.06)	(0.1)	(0.02)	(0.17)	(0.04)	(0.06)	(0.04)	(0.13)	(0.02)	(0.18)	(0.04)

ER+	ER-	k^{train}_{ER+}	SVM						PAM						PLR					
			PA	PA_{ER+}	PA_{ER-}	PV_{ER+}	PV_{ER-}	AUC	PA	PA_{ER+}	PA_{ER-}	PV_{ER+}	PV_{ER-}	AUC	PA	PA_{ER+}	PA_{ER-}	PV_{ER+}	PV_{ER-}	AUC
5	5	0.5	0.79	0.82	0.75	0.78	0.82	0.86	0.72	0.72	0.72	0.74	0.73	0.78	0.79	0.82	0.76	0.78	0.82	0.86
			(0.09)	(0.14)	(0.15)	(0.1)	(0.11)	(0.08)	(0.12)	(0.17)	(0.2)	(0.14)	(0.13)	(0.13)	(0.09)	(0.14)	(0.15)	(0.1)	(0.11)	(0.09)
10		0.67	0.79	0.91	0.66	0.85	0.81	0.87	0.76	0.92	0.6	0.83	0.82	0.84	0.77	0.94	0.59	0.83	0.84	0.87
			(0.09)	(0.08)	(0.17)	(0.06)	(0.14)	(0.07)	(0.1)	(0.08)	(0.22)	(0.08)	(0.16)	(0.09)	(0.09)	(0.06)	(0.19)	(0.06)	(0.14)	(0.07)
20		0.8	0.77	0.94	0.6	0.91	0.76	0.89	0.77	0.94	0.6	0.91	0.77	0.88	0.71	0.97	0.46	0.88	0.82	0.88
			(0.09)	(0.06)	(0.18)	(0.04)	(0.19)	(0.06)	(0.1)	(0.06)	(0.21)	(0.04)	(0.19)	(0.07)	(0.09)	(0.04)	(0.19)	(0.04)	(0.21)	(0.06)
30		0.86	0.74	0.95	0.53	0.93	0.72	0.88	0.75	0.94	0.56	0.93	0.72	0.88	0.67	0.98	0.37	0.9	0.82	0.88
			(0.08)	(0.06)	(0.17)	(0.03)	(0.24)	(0.05)	(0.09)	(0.06)	(0.19)	(0.03)	(0.24)	(0.06)	(0.09)	(0.04)	(0.18)	(0.02)	(0.25)	(0.06)
45		0.9	0.73	0.96	0.49	0.94	0.72	0.88	0.74	0.95	0.53	0.95	0.69	0.89	0.64	0.98	0.3	0.93	0.85	0.89
			(0.08)	(0.05)	(0.17)	(0.02)	(0.29)	(0.05)	(0.08)	(0.05)	(0.18)	(0.02)	(0.28)	(0.05)	(0.08)	(0.03)	(0.16)	(0.02)	(0.28)	(0.05)
10	10	0.5	0.84	0.87	0.82	0.83	0.87	0.91	0.83	0.83	0.83	0.83	0.84	0.89	0.84	0.85	0.82	0.83	0.86	0.9
			(0.05)	(0.09)	(0.09)	(0.06)	(0.08)	(0.04)	(0.07)	(0.11)	(0.1)	(0.07)	(0.09)	(0.06)	(0.06)	(0.1)	(0.09)	(0.06)	(0.08)	(0.04)
20		0.67	0.85	0.9	0.81	0.91	0.82	0.91	0.85	0.9	0.81	0.91	0.82	0.91	0.84	0.92	0.75	0.88	0.84	0.9
			(0.05)	(0.07)	(0.09)	(0.04)	(0.11)	(0.04)	(0.05)	(0.07)	(0.1)	(0.04)	(0.11)	(0.05)	(0.06)	(0.07)	(0.12)	(0.05)	(0.11)	(0.04)
30		0.75	0.85	0.92	0.79	0.93	0.79	0.91	0.85	0.91	0.79	0.93	0.78	0.91	0.82	0.95	0.69	0.9	0.83	0.91
			(0.05)	(0.06)	(0.1)	(0.03)	(0.13)	(0.04)	(0.05)	(0.07)	(0.11)	(0.03)	(0.14)	(0.03)	(0.06)	(0.05)	(0.13)	(0.04)	(0.13)	(0.03)
45		0.82	0.85	0.93	0.77	0.95	0.74	0.91	0.85	0.93	0.78	0.95	0.74	0.92	0.79	0.96	0.61	0.92	0.82	0.91
			(0.05)	(0.06)	(0.1)	(0.02)	(0.17)	(0.04)	(0.06)	(0.06)	(0.11)	(0.02)	(0.17)	(0.04)	(0.07)	(0.04)	(0.13)	(0.03)	(0.18)	(0.04)

Table 1: Predictive accuracy (PA), ER+ PA (PA_{ER+}), ER- PA (PA_{ER-}), predictive values for class ER+ and ER- (PV_{ER+}, PV_{ER-}) and area under the ROC curve (AUC) for different proportions of ER+ samples in the training set (k^{train}_{ER+}). Test set was balanced and contained 20 samples from each class.
