# Supplementary material for: Class prediction for high-dimensional class-imbalanced data
Source: BMC Bioinformatics. 2010 Oct 20;11:523. doi: 10.1186/1471-2105-11-523 (PMC3098087; doi:10.1186/1471-2105-11-523)
Supplement: Additional file 14 — Behavior of the nine classifiers for the prediction of ER status using multiple down-sizing. The table shows, for all the classifiers, the predictive accuracies obtained in Figure 6 using multiple down-sizing, together with the predictive values and AUC. [file 1471-2105-11-523-S14.PDF]

ER+	ER-	k_{ER+}^{train}	1-NN PA	PA_{ER+}	PA_{ER-}	PV_{ER+}	PV_{ER-}	AUC	3-NN PA	PA_{ER+}	PA_{ER-}	PV_{ER+}	PV_{ER-}	AUC	5-NN PA	PA_{ER+}	PA_{ER-}	PV_{ER+}	PV_{ER-}	AUC
10	5	0.67	0.81	0.85	0.77	0.88	0.75	0.88	0.81	0.85	0.78	0.89	0.74	0.88	0.81	0.84	0.78	0.89	0.74	0.89
			(0.07)	(0.11)	(0.13)	(0.05)	(0.14)	(0.06)	(0.07)	(0.11)	(0.13)	(0.05)	(0.13)	(0.06)	(0.07)	(0.11)	(0.13)	(0.05)	(0.13)	(0.06)
20		0.8	0.82	0.85	0.79	0.94	0.61	0.89	0.83	0.85	0.8	0.95	0.62	0.89	0.83	0.85	0.8	0.95	0.61	0.9
			(0.07)	(0.1)	(0.11)	(0.03)	(0.17)	(0.05)	(0.07)	(0.1)	(0.11)	(0.03)	(0.16)	(0.05)	(0.07)	(0.1)	(0.12)	(0.03)	(0.17)	(0.05)
30		0.86	0.83	0.85	0.8	0.96	0.54	0.9	0.83	0.85	0.81	0.96	0.54	0.9	0.83	0.85	0.81	0.97	0.53	0.9
			(0.06)	(0.1)	(0.1)	(0.02)	(0.18)	(0.05)	(0.06)	(0.1)	(0.1)	(0.02)	(0.18)	(0.05)	(0.06)	(0.09)	(0.1)	(0.02)	(0.18)	(0.05)
45		0.9	0.83	0.86	0.8	0.97	0.46	0.89	0.83	0.86	0.81	0.98	0.46	0.9	0.83	0.86	0.81	0.98	0.46	0.9
			(0.06)	(0.09)	(0.1)	(0.01)	(0.2)	(0.05)	(0.06)	(0.09)	(0.1)	(0.01)	(0.2)	(0.05)	(0.06)	(0.09)	(0.1)	(0.01)	(0.2)	(0.05)
20	10	0.67	0.85	0.86	0.83	0.91	0.77	0.9	0.86	0.88	0.84	0.92	0.79	0.91	0.86	0.88	0.84	0.92	0.79	0.91
			(0.05)	(0.09)	(0.07)	(0.03)	(0.11)	(0.04)	(0.05)	(0.08)	(0.07)	(0.03)	(0.11)	(0.04)	(0.05)	(0.07)	(0.06)	(0.03)	(0.11)	(0.04)
30		0.75	0.86	0.87	0.84	0.94	0.71	0.91	0.86	0.89	0.84	0.94	0.74	0.91	0.87	0.89	0.84	0.95	0.73	0.91
			(0.04)	(0.08)	(0.07)	(0.02)	(0.13)	(0.04)	(0.04)	(0.07)	(0.06)	(0.02)	(0.13)	(0.04)	(0.04)	(0.07)	(0.06)	(0.02)	(0.13)	(0.04)
45		0.82	0.86	0.87	0.84	0.96	0.64	0.91	0.86	0.89	0.84	0.96	0.66	0.92	0.86	0.88	0.84	0.96	0.65	0.92
			(0.04)	(0.08)	(0.07)	(0.02)	(0.15)	(0.04)	(0.04)	(0.07)	(0.06)	(0.01)	(0.15)	(0.04)	(0.04)	(0.07)	(0.06)	(0.01)	(0.15)	(0.04)

ER+	ER-	k_{ER+}^{train}	DLDA PA	PA_{ER+}	PA_{ER-}	PV_{ER+}	PV_{ER-}	AUC	DQDA PA	PA_{ER+}	PA_{ER-}	PV_{ER+}	PV_{ER-}	AUC	RF PA	PA_{ER+}	PA_{ER-}	PV_{ER+}	PV_{ER-}	AUC
10	5	0.67	0.77	0.82	0.71	0.86	0.7	0.85	0.77	0.82	0.72	0.86	0.71	0.86	0.77	0.83	0.72	0.86	0.7	0.85
			(0.08)	(0.12)	(0.15)	(0.06)	(0.14)	(0.07)	(0.09)	(0.14)	(0.15)	(0.06)	(0.16)	(0.08)	(0.08)	(0.12)	(0.15)	(0.06)	(0.15)	(0.07)
20		0.8	0.78	0.83	0.74	0.93	0.57	0.86	0.78	0.82	0.74	0.93	0.58	0.87	0.79	0.83	0.75	0.93	0.57	0.86
			(0.08)	(0.11)	(0.14)	(0.03)	(0.18)	(0.07)	(0.07)	(0.14)	(0.14)	(0.03)	(0.2)	(0.07)	(0.08)	(0.11)	(0.13)	(0.03)	(0.18)	(0.07)
30		0.86	0.79	0.84	0.74	0.95	0.49	0.87	0.79	0.83	0.75	0.95	0.51	0.87	0.8	0.84	0.75	0.95	0.5	0.87
			(0.08)	(0.11)	(0.14)	(0.02)	(0.19)	(0.06)	(0.07)	(0.13)	(0.13)	(0.02)	(0.21)	(0.06)	(0.08)	(0.11)	(0.13)	(0.02)	(0.19)	(0.06)
45		0.9	0.79	0.84	0.75	0.97	0.41	0.87	0.8	0.83	0.76	0.97	0.44	0.88	0.8	0.84	0.75	0.97	0.42	0.87
			(0.07)	(0.1)	(0.12)	(0.01)	(0.2)	(0.06)	(0.07)	(0.13)	(0.12)	(0.01)	(0.23)	(0.07)	(0.07)	(0.1)	(0.12)	(0.01)	(0.2)	(0.06)
20	10	0.67	0.84	0.85	0.82	0.91	0.75	0.9	0.84	0.86	0.83	0.91	0.76	0.9	0.84	0.86	0.82	0.91	0.76	0.9
			(0.05)	(0.08)	(0.08)	(0.04)	(0.11)	(0.04)	(0.05)	(0.09)	(0.09)	(0.04)	(0.11)	(0.04)	(0.05)	(0.08)	(0.08)	(0.04)	(0.11)	(0.04)
30		0.75	0.84	0.86	0.83	0.94	0.68	0.9	0.85	0.86	0.84	0.94	0.7	0.91	0.85	0.86	0.83	0.94	0.69	0.9
			(0.05)	(0.08)	(0.08)	(0.03)	(0.13)	(0.04)	(0.05)	(0.09)	(0.08)	(0.03)	(0.14)	(0.04)	(0.05)	(0.08)	(0.08)	(0.03)	(0.13)	(0.04)
45		0.82	0.84	0.86	0.82	0.96	0.6	0.91	0.85	0.86	0.83	0.96	0.62	0.91	0.84	0.87	0.82	0.96	0.61	0.91
			(0.05)	(0.08)	(0.08)	(0.02)	(0.15)	(0.04)	(0.05)	(0.09)	(0.08)	(0.02)	(0.17)	(0.04)	(0.05)	(0.08)	(0.08)	(0.02)	(0.15)	(0.04)

ER+	ER-	k_{ER+}^{train}	SVM PA	PA_{ER+}	PA_{ER-}	PV_{ER+}	PV_{ER-}	AUC	PAM PA	PA_{ER+}	PA_{ER-}	PV_{ER+}	PV_{ER-}	AUC	PLR PA	PA_{ER+}	PA_{ER-}	PV_{ER+}	PV_{ER-}	AUC
10	5	0.67	0.81	0.85	0.78	0.89	0.75	0.88	0.79	0.8	0.78	0.88	0.68	0.87	0.82	0.85	0.78	0.89	0.75	0.88
			(0.07)	(0.11)	(0.12)	(0.05)	(0.14)	(0.06)	(0.09)	(0.13)	(0.15)	(0.07)	(0.14)	(0.07)	(0.07)	(0.11)	(0.12)	(0.05)	(0.13)	(0.06)
20		0.8	0.83	0.85	0.8	0.95	0.62	0.89	0.81	0.81	0.8	0.94	0.56	0.89	0.83	0.85	0.8	0.95	0.61	0.89
			(0.07)	(0.1)	(0.11)	(0.03)	(0.16)	(0.05)	(0.08)	(0.12)	(0.12)	(0.03)	(0.17)	(0.06)	(0.06)	(0.1)	(0.11)	(0.03)	(0.16)	(0.05)
30		0.86	0.83	0.85	0.81	0.96	0.54	0.9	0.82	0.83	0.81	0.96	0.49	0.9	0.83	0.85	0.81	0.96	0.54	0.9
			(0.06)	(0.1)	(0.1)	(0.02)	(0.18)	(0.05)	(0.07)	(0.11)	(0.12)	(0.02)	(0.17)	(0.05)	(0.06)	(0.1)	(0.1)	(0.02)	(0.18)	(0.05)
45		0.9	0.83	0.86	0.81	0.98	0.46	0.9	0.83	0.83	0.82	0.98	0.42	0.9	0.84	0.86	0.81	0.98	0.46	0.9
			(0.06)	(0.09)	(0.09)	(0.01)	(0.2)	(0.05)	(0.07)	(0.1)	(0.11)	(0.01)	(0.19)	(0.05)	(0.06)	(0.09)	(0.09)	(0.01)	(0.2)	(0.05)
20	10	0.67	0.86	0.88	0.84	0.92	0.79	0.91	0.85	0.86	0.84	0.92	0.77	0.91	0.85	0.87	0.84	0.92	0.78	0.9
			(0.05)	(0.07)	(0.06)	(0.03)	(0.11)	(0.04)	(0.05)	(0.08)	(0.07)	(0.03)	(0.1)	(0.04)	(0.05)	(0.08)	(0.06)	(0.03)	(0.11)	(0.04)
30		0.75	0.87	0.89	0.85	0.95	0.74	0.91	0.86	0.87	0.85	0.95	0.71	0.91	0.86	0.87	0.85	0.94	0.72	0.91
			(0.04)	(0.07)	(0.06)	(0.02)	(0.13)	(0.04)	(0.05)	(0.08)	(0.06)	(0.02)	(0.13)	(0.04)	(0.05)	(0.08)	(0.07)	(0.02)	(0.14)	(0.04)
45		0.82	0.86	0.89	0.84	0.96	0.66	0.91	0.86	0.87	0.85	0.96	0.63	0.92	0.86	0.87	0.84	0.96	0.63	0.92
			(0.04)	(0.07)	(0.06)	(0.01)	(0.15)	(0.04)	(0.04)	(0.08)	(0.06)	(0.01)	(0.15)	(0.04)	(0.04)	(0.08)	(0.06)	(0.01)	(0.16)	(0.04)

Table 1: Predictive accuracy (PA), class 1 PA (PA_1), class 2 PA (PA_2), positive and negative predictive value (PV_1, PV_2) and area under the ROC curve (AUC) for different proportions of ER- samples in the training set . Test set was balanced and contained 20 samples from each class. Multiple down-sizing was used to balance class distribution.
